# Fine Particulate Matter, Its Constituents, and Spontaneous Preterm Birth

**DOI:** 10.1001/jamanetworkopen.2024.44593

**Published:** 2024-11-13

**Authors:** Anqi Jiao, Alexa N. Reilly, Tarik Benmarhnia, Yi Sun, Chantal Avila, Vicki Chiu, Jeff Slezak, David A. Sacks, John Molitor, Mengyi Li, Jiu-Chiuan Chen, Jun Wu, Darios Getahun

**Affiliations:** 1 Department of Environmental and Occupational Health, Program in Public Health, University of California, Irvine; 2 Kaiser Permanente Bernard J. Tyson School of Medicine, Pasadena, California; 3 Scripps Institution of Oceanography, University of California, San Diego; 4 Irset Institut de Recherche en Santé, Environnement et Travail, UMR-S 1085, Inserm, University of Rennes, École des Hautes Études en Santé Publique, Rennes, France; 5 Institute of Medical Information, Chinese Academy of Medical Sciences and Peking Union Medical College, Beijing, China; 6 Department of Research and Evaluation, Kaiser Permanente Southern California, Pasadena; 7 Department of Obstetrics and Gynecology, Keck School of Medicine, University of Southern California, Los Angeles; 8 College of Public Health and Human Sciences, Oregon State University, Corvallis; 9 Department of Population and Public Health Sciences, University of Southern California, Los Angeles; 10 Department of Health Systems Science, Kaiser Permanente Bernard J. Tyson School of Medicine, Pasadena, California

## Abstract

**Importance:**

The associations of exposure to fine particulate matter (PM_2.5_) and its constituents with spontaneous preterm birth (sPTB) remain understudied. Identifying subpopulations at increased risk characterized by socioeconomic status and other environmental factors is critical for targeted interventions.

**Objective:**

To examine associations of PM_2.5_ and its constituents with sPTB.

**Design, Setting, and Participants:**

This population-based retrospective cohort study was conducted from 2008 to 2018 within a large integrated health care system, Kaiser Permanente Southern California. Singleton live births with recorded residential information of pregnant individuals during pregnancy were included. Data were analyzed from December 2023 to March 2024.

**Exposures:**

Daily total PM_2.5_ concentrations and monthly data on 5 PM_2.5_ constituents (sulfate, nitrate, ammonium, organic matter, and black carbon) in California were assessed, and mean exposures to these pollutants during pregnancy and by trimester were calculated. Exposures to total green space, trees, low-lying vegetation, and grass were estimated using street view images. Wildfire-related exposure was measured by the mean concentration of wildfire-specific PM_2.5_ during pregnancy. Additionally, the mean exposure to daily maximum temperature during pregnancy was calculated.

**Main Outcomes and Measures:**

The primary outcome was sPTB identified through a natural language processing algorithm. Discrete-time survival models were used to estimate associations of total PM_2.5_ concentration and its 5 constituents with sPTB. Interaction terms were used to examine the effect modification by race and ethnicity, educational attainment, household income, and exposures to green space, wildfire smoke, and temperature.

**Results:**

Among 409 037 births (mean [SD] age of mothers at delivery, 30.3 [5.8] years), there were positive associations of PM_2.5_, black carbon, nitrate, and sulfate with sPTB. Adjusted odds ratios (aORs) per IQR increase were 1.15 (95% CI, 1.12-1.18; *P* < .001) for PM_2.5_ (IQR, 2.76 μg/m^3^), 1.15 (95% CI, 1.11-1.20; *P* < .001) for black carbon (IQR, 1.05 μg/m^3^), 1.09 (95% CI, 1.06-1.13; *P* < .001) for nitrate (IQR, 0.93 μg/m^3^), and 1.06 (95% CI, 1.03-1.09; *P* < .001) for sulfate (IQR, 0.40 μg/m^3^) over the entire pregnancy. The second trimester was the most susceptible window; for example, aORs for total PM_2.5_ concentration were 1.07 (95% CI, 1.05-1.09; *P* < .001) in the first, 1.10 (95% CI, 1.08-1.12; *P* < .001) in the second, and 1.09 (95% CI, 1.07-1.11; *P* < .001) in the third trimester. Significantly higher aORs were observed among individuals with lower educational attainment (eg, less than college: aOR, 1.16; 95% CI, 1.12-1.21 vs college [≥4 years]: aOR, 1.10; 95% CI, 1.06-1.14; *P* = .03) or income (<50th percentile: aOR, 1.17; 95% CI, 1.14-1.21 vs ≥50th percentile: aOR, 1.12; 95% CI, 1.09-1.16; *P* = .02) or who were exposed to limited green space (<50th percentile: aOR, 1.19; 95% CI, 1.15-1.23 vs ≥50th percentile: aOR, 1.12; 95% CI, 1.09-1.15; *P* = .003), more wildfire smoke (≥50th percentile: aOR, 1.19; 95% CI, 1.16-1.23 vs <50th percentile: aOR, 1.13; 95% CI, 1.09-1.16; *P* = .009), or extreme heat (aOR, 1.51; 95% CI, 1.42-1.59 vs mild temperature: aOR, 1.11; 95% CI, 1.09-1.14; *P* < .001).

**Conclusions and Relevance:**

In this study, exposures to PM_2.5_ and specific PM_2.5_ constituents during pregnancy were associated with increased odds of sPTB. Socioeconomic status and other environmental exposures modified this association.

## Key Points

**Question:**

Is exposure to fine particulate matter (PM_2.5_) and its constituents associated with spontaneous preterm birth (sPTB), and do socioeconomic status and other environmental exposures modify the association?

**Findings:**

This cohort study of 409 037 births in Southern California found positive associations of PM_2.5_ and its constituents with sPTB. Individuals with lower socioeconomic status or who were exposed to limited green space, more wildfire smoke, or extreme heat had increased risk of sPTB associated with PM_2.5_.

**Meaning:**

This study found that increased ambient PM_2.5_ exposure was associated with higher odds of sPTB, with socioeconomic and other environmental factors potentially modifying this association.

## Teaser

This cohort study investigates the association of fine particulate matter and its constituents with odds of spontaneous preterm birth among members of a large health care system in Southern California.

## Introduction

Preterm birth (PTB) is an important obstetrical event complicating approximately 11% of births worldwide and a leading cause of mortality in children younger than 5 years.^1,2,3^ It can be commonly categorized into spontaneous PTB (sPTB; approximately 60%-70% of all PTBs) and iatrogenic PTB (iPTB; approximately 30%-40% of all PTBs) based on different underlying mechanisms.^4,5^ Medical interventions, including labor induction or prelabor cesarean delivery, can result in iPTB, typically due to conditions that threaten maternal or fetal well-being, such as preeclampsia or eclampsia, placental abruption, or intrauterine growth restriction.^6,7^ Preterm births that follow spontaneous labor or preterm premature rupture of membranes are called sPTB, which is considered a complex syndrome resulting from 4 major pathogenic pathways: infection or inflammation, decidual hemorrhage, uterine overdistension, and premature activation of the maternal or fetal hypothalamic-pituitary-adrenal axis due to stress.^4,8,9^ The prediction and prevention of sPTB remain challenging because causes leading to the disruption of uterine quiescence and cervical changes (with or without rupture of membranes) are still not fully understood.^10,11^ As the major source of prematurity in contemporary obstetrics, sPTB has a significant impact on neonatal morbidity and mortality rates. Previous research has highlighted the importance of identifying and using risk factors associated with sPTB to inform early interventions and has associated various sociodemographic, nutritional, environmental, and genetic factors with the risk of sPTB.^10,12,13,14^

Exposure to PM_2.5_ and its components (eg, black carbon, nitrate, and sulfate) has been associated with several pathophysiological pathways, such as oxidative stress, inflammation, and activation of the hypothalamic-pituitary-adrenal axis,^15,16,17,18,19^ which may directly lead to sPTB. However, studies on PM_2.5_ exposure and sPTB remain limited^20,21,22,23,24,25^ given that differentiating PTB subtypes in previous investigations has been challenging owing to the lack of medical records for information on preterm labor, rupture of membranes, or cervical incompetence.^25,26^ In addition, many studies may lack sufficient cases for a precise analysis of sPTB, resulting in reduced statistical power when focusing exclusively on this outcome.^27^ Given that ambient PM_2.5_ has been identified as a leading environmental risk factor for various health outcomes, it is crucial to understand its association with the risk of sPTB based on research with a relatively large sample size, which can offer population-based insights into the prevention of sPTB. Furthermore, the chemical composition of PM_2.5_ can vary by region due to different pollution sources, and this variation contributes to different toxic effects associated with PM_2.5_ and discrepancies across studies. Examining the association of specific PM_2.5_ constituents with sPTB may help enhance the understanding of PM_2.5_ toxic effects and provide a reference for local emission control.^28,29^

Existing literature has reported persistent socioeconomic, racial, and ethnic disparities in the rate of PTB in the US, where significantly higher rates were found among Black individuals and those with poverty or limited education.^1,5,30^ Individuals with a lower socioeconomic level may experience greater social stressors and underlying health problems, thereby increasing their level of health risks associated with air pollution.^31^ Investigating how these factors may modify the risk of sPTB associated with PM_2.5_ exposure may help identify populations at increased risk and promote health equity. Moreover, it has been suggested that green space exposure is associated with reduced health risks associated with air pollution^32,33^; however, its role in modifying the association with pregnancy outcomes has been less studied.^34,35^ Most studies used the normalized difference vegetation index (NDVI) to measure total green space exposure without distinguishing vegetation types.^26,32,36^ Given that trees and other vegetation (eg, bushes and grass) have been associated with health benefits through different pathways,^37,38,39^ identifying the effect modification by vegetation type may help establish a more effective strategy for mitigating health risks associated with PM_2.5_. In addition, climate change is exacerbating health-threatening conditions, such as extreme heat and wildfires, which may particularly affect pregnant individuals owing to the physiological and psychological changes during pregnancy.^40,41^ Examining the effect modification of these climate-sensitive exposures on the association between PM_2.5_ levels and sPTB may raise awareness and encourage the adoption of self-protection measures in a changing climate.

We conducted a retrospective cohort study in Southern California to examine associations of exposures to total PM_2.5_ and PM_2.5_ constituents during pregnancy with sPTB. Furthermore, we examined the effect modification by race and ethnicity, socioeconomic status, and other environmental exposures (ie, green space, wildfire smoke, and temperature).

## Methods

### Study Population

We identified 429 839 pregnancies with delivery of singleton live births from January 1, 2008, to December 31, 2018, at Kaiser Permanente Southern California health care system (KPSC) (eFigure 1 and eMethods in Supplement 1). Detailed information for each pregnancy was provided in KPSC electronic health records, including sociodemographic characteristics, medical and obstetric histories, birth records, residential histories, and health-related behaviors. Data on race and ethnicity were based on a combination of administrative and patient self-reports,^42,43^ and we reported the data given varying rates of sPTB across different racial and ethnic groups.^4^ Race and ethnicity categories included Asian, Black, Hispanic, non-Hispanic White, and other (including American Indian or Alaska Native, Pacific Islander, and multiple races or ethnicities). Race and ethnicity were obtained in the same question. This study followed the Strengthening the Reporting of Observational Studies in Epidemiology (STROBE) reporting guideline and was approved by the Institutional Review Boards of KPSC and the University of California, Irvine, with an exemption for informed consent because the research was considered minimal risk for participants.

### Outcome Ascertainment

Preterm birth was defined as a live birth that occurs after 20 completed weeks and before 37 completed weeks of gestation. Gestational age was primarily estimated by first-trimester ultrasonography, and for a small minority, by the last menstrual period with confirmation from second-trimester ultrasonography (eMethods in Supplement 1).^31^ We used a natural language processing algorithm to extract information on preterm labor visits from electronic health records.^44^ The algorithm found key terms related to preterm labor triage and evaluation, such as terms referring to fetal fibronectin tests and transvaginal ultrasonography evaluation of cervical length.^4,45,46,47^ Its performance has been validated with a 97% positive predictive value.^44^ To ascertain sPTB, we first identified all preterm labor visits. Then, we defined sPTB as a preterm delivery that follows the spontaneous onset of labor, is not indicated by concomitant pregnancy complications, and occurs within 7 days of the last preterm labor visit.^48,49,50^ All remaining PTBs with medical indications, such as preeclampsia or eclampsia, were grouped as iPTBs.

### Air Pollution Exposure Assessment

We obtained daily total PM_2.5_ concentrations during 2007 to 2018 at the census tract level from a validated ensemble model (eFigures 2 and 3 in Supplement 1),^51^ which incorporated multiple machine-learning algorithms with various explanatory variables. Then, we obtained monthly concentrations of 5 PM_2.5_ constituents (sulfate, nitrate, ammonium, organic matter, and black carbon) during 2007 to 2017^29,52,53^ from the publicly available outputs of a geoscience-derived model at a spatial resolution of 1 km × 1 km.^54,55^ We calculated mean exposures to total PM_2.5_ and PM_2.5_ constituents during the entire pregnancy and in each trimester for each individual based on their residential history and geocoded addresses during pregnancy. Pregnancies with inadequate residential data (<75% of completeness during pregnancy) were excluded (20 802 of 429 839 pregnancies [4.84%]).^31^ The performance of these 2 models and the exclusion criteria are detailed in the eMethods in Supplement 1.

To examine the effect modification by wildfire-related exposure, we estimated the mean exposure to wildfire-specific PM_2.5_ during the entire pregnancy based on the ensemble model described previously.^51,56^ Briefly, nonwildfire PM_2.5_ concentrations that would have been observed if there had been no wildfires were estimated by implementing a multiple imputation approach. Wildfire-specific PM_2.5_ concentrations were estimated as the difference between total and nonwildfire PM_2.5_ concentrations.

### Green Space Exposure Assessment

We obtained street view green space exposure from a validated machine-learning model.^38,57^ We requested high-resolution street view images from Microsoft Bing Maps Application Programming Interface and estimated the proportion of greenery pixels in each image. We distinguished 3 types of vegetation, including trees, low-lying vegetation (eg, shrubs and bushes), and grass. Exposures to total green space and each type of vegetation were estimated by finding the mean proportion of corresponding greenery pixels in all street view images within a 1 km radius surrounding the residential area at delivery. Further details can be found in the eMethods in Supplement 1.

### Temperature Exposure Assessment

We obtained daily maximum temperature data for Southern California from 2007 to 2018 at a 4 km × 4 km resolution from the gridMET dataset.^58,59^ We calculated the mean exposure to daily maximum temperature during pregnancy for each individual based on their geocoded home addresses and accounting for residential mobility. The exposure was further categorized as extreme cold (<10th percentile), mild temperature (10th to 90th percentile), and extreme heat (≥90th percentile).^60,61,62^

### Statistical Analysis

We provide summary statistics of sociodemographics and environmental exposures of the study population. We measured correlations between environmental exposures using Pearson correlation coefficients (eResults and eTables 1 and 2 in Supplement 1). We estimated associations of exposures to total PM_2.5_ and PM_2.5_ constituents with sPTB in the entire pregnancy and by trimester. Discrete-time survival models with logit link^63,64,65^ were applied to accommodate varying lengths of gestations,^66,67,68^ and births after 37 completed weeks of gestation were censored. We applied a quantile-based *g* computation approach that can leverage correlations among exposures^69,70^ to examine the joint association of different PM_2.5_ constituents as a mixture. Constituents associated with sPTB in the main model were further included in the mixture analysis to quantify their contributions to the overall association.^29,53^ We fitted the county of residence as a random effect to account for potential spatial clustering^29,52,53^ and adjusted for important confounders a priori based on existing studies on air pollution and PTB subtypes^22,23,24^ and literature on risk factors associated with sPTB,^5,9,13,14^ including age, race and ethnicity, educational attainment, median household income, prepregnancy body mass index (calculated as weight in kilograms divided by height in meters squared), season of conception, and year of delivery. We considered more confounders (eg, temperature, insurance type, parity, smoking status, and some preexisting medical conditions [ie, diabetes and chronic hypertension]) in the sensitivity analysis to check the robustness of our results. We reported odds ratios (ORs) of sPTB with corresponding 95% CIs per IQR increase in each exposure.

We examined the effect modification of several factors on the association between PM_2.5_ and sPTB by fitting separate models with interaction terms and adjusting each model for all covariates specified in the main analysis and the respective effect modifier. These factors included sociodemographic characteristics, such as race and ethnicity, educational attainment, and median household income. We also considered exposures to various types of green space (ie, total green space, trees, low-lying vegetation, and grass), wildfire smoke, and temperature. To quantify the effect modification by wildfire smoke exposure, we examined its interaction with nonwildfire PM_2.5_ exposure instead of the total PM_2.5_ exposure.

To examine whether associations of total PM_2.5_ concentration with sPTB and iPTB were different, we applied iPTB as a secondary outcome. In addition, we conducted sensitivity analyses to evaluate the robustness of our findings, such as restricting the population to consider only the first birth of each individual in the cohort, adjusting for other ambient pollutants (eg, nitrogen dioxide and ozone), estimating PM_2.5_ exposure based on other data sources, estimating green space exposure based on NDVI and tree canopy cover measurements, and applying propensity score matching (eMethods in Supplement 1). We also report risk differences as an absolute measure of association.^71,72,73^ A 2-sided *P* value <.05 was considered statistically significant. Analyses were completed using SAS statistical software version 9.4 (SAS Institute) and R statistical software version 4.1.3 (R Project for Statistical Computing). Adjustment for multiple comparisons was not made for the secondary outcome or sensitivity analyses, and those results should be interpreted as exploratory. Data were analyzed from December 2023 to March 2024.

## Results

We included 409 037 singleton live births (mean [SD] age of the study population at delivery, 30.3 [5.8] years; 50 978 among Asian [12.46%], 31 481 among Black [7.70%], 208 615 among Hispanic [51.00%], and 107 237 among White [26.22%] mothers) after excluding 20 802 births with inadequate residential data during pregnancy (Table 1). There were 19 341 sPTBs (4.73%) and 11 254 iPTBs (2.75%). Mothers with sPTB and iPTB were more likely to be older (aged ≥35 years), self-identify as Black or Asian, have a lower educational attainment, be overweight, have pregestational diabetes and hypertension, and have a history of PTB. The mean (SD) level of exposure to total PM_2.5_ during pregnancy was 11.40 (2.34) μg/m^3^ and 11.54 (2.04) μg/m^3^ among all births and sPTBs, respectively (Table 2).

**Table 1.** Study Population Characteristics

Maternal characteristic	Births, No. (%)			
	Total (N = 409 037)	Term births (n = 378 442)	sPTB (n = 19 341)	iPTB (n = 11 254)
Age, y				
<25	78 226 (19.12)	72 633 (19.19)	3592 (18.57)	2001 (17.78)
25-34	242 513 (59.29)	225 772 (59.66)	10 793 (55.80)	5948 (52.85)
≥35	88 298 (21.59)	80 037 (21.15)	4956 (25.62)	3305 (29.37)
Race and ethnicity^a^				
Asian	50 978 (12.46)	46 767 (12.36)	2901 (15.00)	1310 (11.64)
Black	31 481 (7.70)	28 365 (7.50)	1930 (9.98)	1186 (10.54)
Hispanic	208 615 (51.00)	192 646 (50.91)	9944 (51.41)	6025 (53.54)
Non-Hispanic White	107 237 (26.22)	100 766 (26.63)	4069 (21.04)	2402 (21.34)
Other^b^	10 684 (2.61)	9857 (2.60)	497 (2.57)	330 (2.93)
Missing	42 (0.01)	41 (0.01)	0	1 (0.01)
Educational attainment				
Less than college	126 450 (30.91)	116 613 (30.81)	6078 (31.43)	3759 (33.40)
College (<4 y)	127 463 (31.16)	117 387 (31.02)	6302 (32.58)	3774 (33.53)
College (≥4 y) or higher	147 134 (35.97)	137 132 (36.24)	6569 (33.96)	3433 (30.50)
Missing	7990 (1.95)	7310 (1.93)	392 (2.03)	288 (2.56)
Median household income, $				
Mean (SD)	59 771 (21 818)	59 865 (21 819)	59 277 (22 038)	57 472 (21 234)
Missing	1254 (0.31)	1150 (0.30)	67 (0.35)	37 (0.33)
Prepregnancy BMI				
Underweight (<18.5)	9743 (2.38)	8928 (2.36)	557 (2.88)	258 (2.29)
Normal weight (18.5-24.9)	173 391 (42.39)	162 118 (42.84)	7777 (40.21)	3496 (31.06)
Overweight (25.0-29.9)	114 457 (27.98)	105 883 (27.98)	5406 (27.95)	3168 (28.15)
Obesity (≥30.0)	109 297 (26.72)	99 544 (26.30)	5496 (28.42)	4257 (37.83)
Missing	2149 (0.53)	1969 (0.52)	105 (0.54)	75 (0.67)
Season of conception				
Cool (November to April)	208 523 (50.98)	192 898 (50.97)	9978 (51.59)	5647 (50.18)
Warm (May to October)	200 514 (49.02)	185 544 (49.03)	9363 (48.41)	5607 (49.82)
Insurance type				
Medicaid	38 652 (9.45)	35 489 (9.38)	2104 (10.88)	1059 (9.41)
Other	363 469 (88.86)	336 551 (88.93)	16 969 (87.74)	9949 (88.40)
Missing	6916 (1.69)	6402 (1.69)	268 (1.39)	246 (2.19)
Parity				
Primiparous	167 283 (40.90)	154 467 (40.82)	7990 (41.31)	4826 (42.88)
Multiparous	241 212 (58.97)	223 514 (59.06)	11 295 (58.40)	6403 (56.90)
Missing	542 (0.13)	461 (0.12)	56 (0.29)	25 (0.22)
Smoking status				
Never smoker	340 517 (83.25)	315 282 (83.31)	15 995 (82.70)	9240 (82.10)
Past smoker	47 447 (11.60)	43 840 (11.58)	2275 (11.76)	1332 (11.84)
Smoker during pregnancy	21 038 (5.14)	19 292 (5.10)	1069 (5.53)	677 (6.02)
Missing	35 (0.01)	28 (0.01)	2 (0.01)	5 (0.04)
Medical conditions				
Pre-existing diabetes	5424 (1.33)	4244 (1.12)	553 (2.86)	627 (5.57)
Chronic hypertension	13 952 (3.41)	11 157 (2.95)	1107 (5.72)	1688 (15.00)
History of PTB	8819 (2.16)	6683 (1.77)	1403 (7.25)	733 (6.51)

Abbreviations: BMI, body mass index (calculated as weight in kilograms divided by height in meters squared); iPTB, medically indicated preterm birth; PTB, preterm birth; sPTB, spontaneous preterm birth.

^a^ Race and ethnicity data were based on a combination of administrative and patient self-reports. The data were reported given varying risks of sPTB across different racial and ethnic groups.

^b^ Other included American Indian or Alaska Native, Pacific Islander, and multiple races or ethnicities, consolidated owing to a relatively small sample size of each group in the study.

**Table 2.** Environmental Exposures During Entire Pregnancy

Type	Exposure level, mean (SD)			
	Total births (N = 409 037)	Term births (n = 378 442)	sPTB (n = 19 341)	iPTB (n = 11 254)
PM_2.5_ air pollution, μg/m^3^^a^				
Total	11.40 (2.34)	11.39 (2.33)	11.54 (2.04)	11.31 (2.96)
Sulfate	1.28 (0.28)	1.28 (0.28)	1.29 (0.31)	1.26 (0.31)
Nitrate	2.41 (0.65)	2.41 (0.65)	2.43 (0.57)	2.41 (0.80)
Ammonium	0.95 (0.32)	0.95 (0.32)	0.96 (0.30)	0.95 (0.36)
Organic matter	5.39 (1.32)	5.39 (1.32)	5.45 (1.29)	5.35 (1.41)
Black carbon	1.49 (0.62)	1.49 (0.62)	1.54 (0.62)	1.42 (0.62)
Nonwildfire	11.28 (2.31)	11.27 (2.31)	11.43 (2.01)	11.17 (2.91)
Wildfire specific	0.12 (0.14)	0.12 (0.14)	0.11 (0.13)	0.14 (0.19)
Green space, %^b^				
Total	25.27 (3.68)	25.28 (3.69)	25.26 (3.53)	24.98 (3.56)
Trees	15.21 (3.79)	15.22 (3.80)	15.31 (3.66)	14.81 (3.70)
Low-lying vegetation	4.69 (1.36)	4.70 (1.36)	4.64 (1.29)	4.72 (1.47)
Grass	5.36 (1.35)	5.36 (1.35)	5.31 (1.28)	5.45 (1.41)
Temperature exposure, °C^c^	25.18 (2.30)	25.18 (2.27)	25.09 (2.52)	25.26 (2.86)

Abbreviations: iPTB, medically indicated preterm birth; PM_2.5_, particulate matter less than or equal to 2.5 μm; sPTB, spontaneous preterm birth.

^a^ The IQRs of exposures to total PM_2.5_, sulfate, nitrate, ammonium, organic matter, black carbon, nonwildfire PM_2.5_, and wildfire-specific PM_2.5_ were 2.76 μg/m^3^, 0.40 μg/m^3^, 0.93 μg/m^3^, 0.40 μg/m^3^, 1.82 μg/m^3^, 1.05 μg/m^3^, 2.75 μg/m^3^, and 0.15 μg/m^3^, respectively.

^b^ The medians of exposures to total PM_2.5_, wildfire-specific PM_2.5_, total green space, trees, low-lying vegetation, and grass were 11.51 μg/m^3^, 0.065 μg/m^3^, 24.56%, 14.68%, 4.44%, and 5.20%, respectively.

^c^ The mean exposure to daily maximum temperature during pregnancy was assessed. The 10th and 90th percentiles were 22.29 °C and 28.24 °C, respectively.

### Associations of Exposure to Total PM_2.5_ and PM_2.5_ Constituents With sPTB

For associations during the entire pregnancy, the adjusted OR (aOR) per IQR increase (2.76 μg/m^3^) in total PM_2.5_ exposure was 1.15 (95% CI, 1.12-1.18; *P* < .001) (Table 3). Per 1 μg/m^3^ increase, the aOR was 1.05 (95% CI, 1.04-1.06; *P* < .001). Associations were also observed per IQR increase for four PM_2.5_ constituents: sulfate (aOR, 1.06; 95% CI, 1.03-1.09; *P* < .001; IQR, 0.40 μg/m^3^), nitrate (aOR, 1.09; 95% CI, 1.06-1.13; *P* < .001; IQR, 0.93 μg/m^3^), organic matter (aOR, 1.05; 95% CI, 1.02-1.08; *P* < .001; IQR, 1.82 µg/m^3^), and black carbon, which had the highest increase in odds (aOR, 1.15; 95% CI, 1.11-1.20; *P* < .001; IQR, 1.05 μg/m^3^). Consistently higher aORs in association of total PM_2.5_ and PM_2.5_ constituents with sPTB were shown during the second trimester. For example, aORs for total PM_2.5_ concentration were 1.07 (95% CI, 1.05-1.09; *P* < .001) in the first, 1.10 (95% CI, 1.08-1.12; *P* < .001) in the second, and 1.09 (95% CI, 1.07-1.11; *P* < .001) in the third trimester.

**Table 3.** Association Per IQR Increase of PM_2.5_ Exposure With sPTB

PM_2.5_ exposure	sPTB, aOR (95% CI)^a^			
	First trimester	Second trimester	Third trimester	Entire pregnancy
Total	1.07 (1.05-1.09)	1.10 (1.08-1.12)	1.09 (1.07-1.11)	1.15 (1.12-1.18)
Sulfate	1.02 (1.00-1.03)	1.02 (1.01-1.04)	1.00 (0.99-1.01)	1.06 (1.03-1.09)
Nitrate	1.04 (1.01-1.06)	1.06 (1.04-1.09)	1.04 (1.02-1.06)	1.09 (1.06-1.13)
Ammonium	1.01 (0.99-1.03)	1.03 (1.01-1.05)	1.00 (0.98-1.02)	1.03 (1.00-1.06)
Organic matter	1.01 (0.99-1.03)	1.05 (1.03-1.08)	1.03 (1.01-1.05)	1.05 (1.02-1.08)
Black carbon	1.07 (1.04-1.11)	1.13 (1.10-1.17)	1.06 (1.03-1.09)	1.15 (1.11-1.20)

Abbreviations: PM_2.5_, particulate matter less than or equal to 2.5 μm; sPTB, spontaneous preterm birth.

^a^ Models were adjusted for age, race and ethnicity, educational attainment, median household income, prepregnancy body mass index (calculated as weight in kilograms divided by height in meters squared), season of conception, year of delivery, and county of residence.

A quartile increase in the mixture consisting of PM_2.5_ sulfate, nitrate, organic matter, and black carbon during pregnancy was associated with an increase in the odds of sPTB (aOR, 1.09; 95% CI, 1.06-1.13; *P* < .001) (Table 4). PM_2.5_ black carbon, nitrate, and sulfate contributed 37.73%, 34.34%, and 27.93%, respectively, to the positive association.

**Table 4.** Association Between Exposure to PM_2.5_ Mixture During Pregnancy and sPTB Estimated by Quantile-Based *g* Computation

Association	Contribution to association, %	Coefficient, β^a^	Overall coefficient^b^		Overall association^c^	
			β (95% CI)	*P* value	OR (95% CI)	*P* value
Positive association with sPTB			0.09 (0.06-0.12)	<.001	1.09 (1.06-1.13)	<.001
PM_2.5_ sulfate	27.93	0.09				
PM_2.5_ nitrate	34.34					
PM_2.5_ black carbon	37.73					
Negative association with sPTB						
PM_2.5_ organic matter	100^d^	−0.005				

Abbreviations: OR, odds ratio; PM_2.5_, particulate matter less than or equal to 2.5 μm; sPTB, spontaneous preterm birth.

^a^ Models were adjusted for age, race and ethnicity, educational attainment, median household income, prepregnancy body mass index, season of conception, year of delivery, and county of residence.

^b^ The sum of coefficients for associations in positive and negative directions.

^c^ The overall association between exposure to PM_2.5_ mixture and sPTB per quartile increase in PM_2.5_ mixture.

^d^ The contribution was 100% given that this was the only pollutant negatively associated with the outcome in the model.

### Effect Modification by Sociodemographic Characteristics and Other Environmental Exposures

Individuals with lower educational attainment (eg, less than college: aOR, 1.16; 95% CI, 1.12-1.21 vs college [≥4 years]: aOR, 1.10; 95% CI, 1.06-1.14; *P* = .03) or median household income (<50th percentile: aOR, 1.17; 95% CI, 1.14-1.21 vs ≥50th percentile: aOR, 1.12; 95% CI, 1.09-1.16; *P* = .02) had significantly higher aORs in the association of total PM_2.5_ concentration with sPTB (Figure). The increases in odds in the association of total PM_2.5_ concentration with sPTB were significantly higher among mothers with lower exposure to total green space (<50th percentile: aOR, 1.19; 95% CI, 1.15-1.23 vs ≥50th percentile: aOR, 1.12; 95% CI, 1.09-1.15; *P* = .003) and trees (<50th percentile: aOR, 1.19; 95% CI, 1.15-1.22 vs ≥50th percentile: aOR, 1.09; 95% CI, 1.05-1.13; *P* < .001) but lower among those with lower exposure to low-lying vegetation. Furthermore, individuals exposed to more wildfire smoke (≥50th percentile: aOR, 1.19; 95% CI, 1.16-1.23 vs <50th percentile: aOR, 1.13; 95% CI, 1.09-1.16; *P* = .009) and extreme heat (aOR, 1.51; 95% CI, 1.42-1.59 vs mild temperature: aOR, 1.11; 95% CI, 1.09-1.14; *P* < .001) during pregnancy had significantly higher increases in odds of sPTB in the association with PM_2.5_ exposure.

**Figure.**
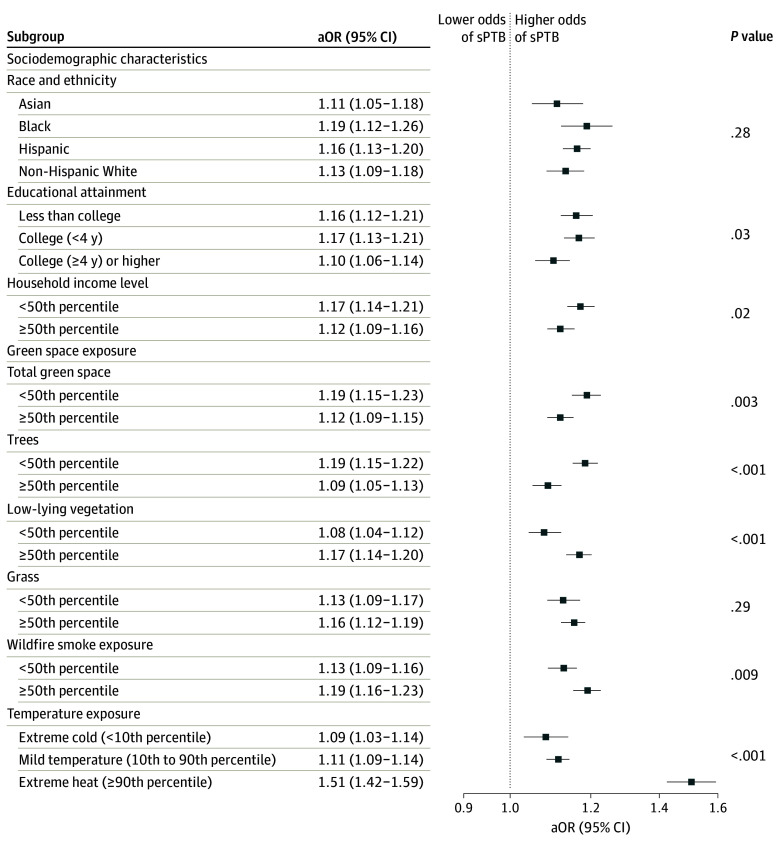
Fine Particulate Matter Exposure During Pregnancy and Odds of Spontaneous Preterm Birth (sPTB) Models were adjusted for age, race and ethnicity, educational attainment, median household income, prepregnancy body mass index (calculated as weight in kilograms divided by height in meters squared), season of conception, year of delivery, county of residence, and respective effect modifier. The *P* value is for the interaction term between fine particulate matter exposure and each effect modifier. aOR indicates adjusted odds ratio.

### Sensitivity Analyses

Results from sensitivity analyses did not change our conclusions (eResults, eFigures 4-6, and eTables 3-7 in Supplement 1). We observed negative associations between PM_2.5_ exposure and iPTB during the entire pregnancy and across 3 trimesters (eResults in Supplement 1). The aOR of iPTB per IQR increase in total PM_2.5_ exposure during pregnancy was 0.81 (95% CI, 0.79-0.83; *P* < .001).

## Discussion

Based on a large and diverse birth cohort, this cohort study found that exposure to total PM_2.5_ during pregnancy was associated with increased odds of sPTB, with the second trimester identified as the most susceptible window. A relatively higher increase in odds in the association between PM_2.5_ black carbon and sPTB was observed among the 5 PM_2.5_ constituents of interest, followed by nitrate and sulfate, while ammonium and organic matter showed minimal increases in odds.

Although prior studies on PM_2.5_ and sPTB were limited, a 2023 study^21^ based on a large birth cohort from 2001 to 2019 in New South Wales, Australia, reported an association between PM_2.5_ exposure and sPTB during pregnancy (hazard ratio per 1 μg/m^3^ increase in PM_2.5_, 1.013; 95% CI, 1.003-1.024). However, the effect size was relatively smaller compared with our results (aOR per 1 μg/m^3^ increase, 1.05; 95% CI, 1.04-1.06; *P* < .001). A study from Shanghai, China,^25^ that examined trimester-specific associations between PM_2.5_ and sPTB reported a positive association in the third trimester. As previously discussed, the underlying mechanisms of the association between PM_2.5_ exposure and sPTB may include an increase in systematic and placental oxidative stress, inflammation, placental DNA methylation, and endocrine disruption, which can lead to placental impairment and affect the structure of chorioamniotic membranes.^15,16,74,75,76,77,78,79^ However, 2 studies from the US reported either null or negative associations of PM_2.5_ with sPTB.^22,23^ Discrepancies between their studies and ours may be due to different study designs, populations, and exposure assessment approaches.

Although iPTB is clinician initiated and occurs without a natural onset of labor, indications for iPTB, such as preeclampsia and eclampsia, have been associated with PM_2.5_ exposure in some studies.^75,80^ Therefore, iPTB may be indirectly associated with air pollution, with some indications potentially lying in the pathway. However, we observed negative associations between PM_2.5_ exposure and iPTB during pregnancy. Similarly, 2 studies conducted in the US^22,23^ observed nonsignificant decreases in the risk of iPTB associated with PM_2.5_ exposure, where 1 of these studies reported negative associations of nitrogen dioxide with iPTB.^22^ Given that iPTB is typically triggered by medical interventions, the rate and timing of such interventions can vary due to differing medical practices among clinicians. If these variations in health care practices correlate with fluctuations in air pollution levels, they could confound the association between environmental pollutants and iPTB.^22^ Previous literature has suggested that the pattern of iPTB may be influenced by complex socioeconomic factors interacting with the medical care system.^27^ Health care facilities located in areas with high socioeconomic status and low air pollution may tend to prescribe iPTB more often to patients at risk.^81^ Therefore, the observed negative association may be a consequence of residual confounding due to artificial or external factors not adjusted for in our analysis.^4,22^ Different results for sPTB and iPTB underscore a need for differentiating PTB subtypes in future studies.

The presence or absence of labor is critical to distinguish between sPTB and iPTB. Some previous studies on environmental exposures and PTB failed to isolate sPTB^82,83^ or identified sPTB by excluding all cesarean deliveries or by relying on noninduced labor owing to the unavailability of labor information.^84,85^ These approaches are likely to misclassify PTB subtypes and bias findings given that cesarean delivery can occur for medically indicated or elective reasons or occur after a trial of labor and the terms *induction of labor* and *augmentation of insufficient labor* are sometimes used interchangeably. In addition, some studies^22,86,87^ have used *International Classification of Diseases, Ninth Revision *(*ICD-9*) codes as proxies to distinguish among PTB subtypes given that indicators for the presence of labor were not readily available. These studies considered cesarean deliveries (without codes indicating labor or spontaneous delivery), artificial rupture of membranes, and induction of labor as iPTB; all other cases were classified as sPTB, which may still lead to misclassification given that whether labor was present or absent was inferred from these *ICD* code proxies. Our study identified individuals triaged for preterm labor evaluation and implemented an extensive natural language processing algorithm to accurately capture indicators associated with impending sPTB based on a rich clinical database that can provide information on preterm labor symptoms, fetal fibronectin tests, and cervical length measurements. Therefore, we provided a more robust ascertainment of pregnancies resulting in sPTB based on the presence or absence of labor with corresponding clinical symptoms and restricting the interval between the onset of labor and the time of delivery.

We observed that individuals without a 4-year college degree or with lower household incomes were at increased risk of sPTB associated with PM_2.5_ exposure, highlighting the significant issues of health inequity among pregnant individuals. Individuals with lower socioeconomic status may have more stressful social experiences and live in hazardous physical environments.^88,89,90^ In addition, they may have poorer health status, less access to medical resources, or more adverse health-related behaviors before and during pregnancy.^30,91,92^ Targeted and preventive public health interventions among these subpopulations with high risk may be critical for minimizing the burden of sPTB.

To offer insights for prospective mitigation actions, we examined the effect modification by street view green space exposure. Compared with satellite-based data, street-level green space may better reflect actual exposure and illustrate exposure pathways.^93,94^ Our study found that more total green space and trees, rather than low-lying vegetation or grass, modified the association between PM_2.5_ and sPTB to have a smaller increase in odds. To increase the comparability of our study with others, we conducted sensitivity analyses for NDVI and tree canopy exposure, and the results also supported these findings. Some studies have shown more benefits associated with urban trees than other vegetation types.^37,38,39^ Although trees can increase the deposition of particles, they may also inhibit particle dispersion near emission sources and deteriorate air quality in the area.^95^ More in-depth studies considering local conditions and different plant species may help maximize the benefits of green space.^33,96^ Moreover, our findings suggest that people exposed to more wildfire smoke or extreme heat during pregnancy may experience a double jeopardy. Some studies also reported a higher increase in risk of respiratory diseases associated with ambient PM_2.5_ among fire-affected areas or in fire seasons.^97,98,99,100^ The mental stress associated with wildfire events and the health shock induced by sporadic extreme pollution events during pregnancy may both contribute to increased risk.^101,102^

To our knowledge, this is the first study that examined associations of PM_2.5_ and its constituent concentrations with sPTB by applying a natural language processing algorithm to define PTB subtypes reliably. In addition, we examined the effect modification by specific types of green space to inform a more efficient mitigation strategy. Furthermore, our study benefited from a large sample size with a socioeconomically diverse population, a comprehensive clinical database, and a more accurate exposure assessment based on detailed information on prenatal residential mobility.

### Limitations

This study has some limitations. First, exposure misclassification was inevitable given that we estimated individual exposure to PM_2.5_ based on census tract–level data and did not consider personal time-activity patterns (eg, time indoors or in the workplace) owing to data unavailability, which can bias associations in either direction. In sensitivity analyses, we examined associations of PM_2.5_ using data from other sources^55,103^ and found similar results, indicating the robustness of our conclusions. Second, we considered only 5 major PM_2.5_ constituents owing to data unavailability. Associations of other elements attached to PM_2.5_ (eg, polycyclic aromatic hydrocarbons) may deserve future investigations. Additionally, we obtained only monthly data on PM_2.5_ constituents, which may not be accurate enough and may lead to exposure misclassification. Third, street view green space data were considered spatial snapshot data that cannot capture temporal variations, which may lead to exposure misclassification and bias associations in either direction. Fourth, we did not assess different clinical phenotypes of sPTB.^20,104^ Future studies with data to distinguish various phenotypes may promote a deeper understanding of the biological mechanisms.

## Conclusions

This cohort study found that more exposure to ambient PM_2.5_ during pregnancy was associated with increased odds of sPTB. Individuals with lower socioeconomic status and those exposed to more wildfire smoke or extreme heat during pregnancy were found to be at greater risk. More green space exposure, especially trees, may modify the association of PM_2.5_ concentration with sPTB, with smaller increases in odds.

## Notes

Supplement 1.
eMethods.

eResults.
**eFigure 1. **The composition of the Kaiser Permanente Southern California pregnancy cohort from 2008 to 2018 and the population selection process **eFigure 2.** The temporal trend of daily mean concentrations of total, nonwildfire, and wildfire-specific PM_2.5_ across California from 2007 to 2018 based on the census tract–level data **eFigure 3.** The mean exposure to total PM_2.5_, nonwildfire PM_2.5_, and wildfire-specific PM_2.5_ during pregnancy for the study population **eFigure 4.** The adjusted odds ratios (ORs) with 95% CIs of spontaneous preterm birth (sPTB) associated with exposure to total PM_2.5_ during pregnancy among subgroups stratified by street view green space exposure in 200 m and 500 m buffers **eFigure 5.** The adjusted odds ratios (ORs) with 95% CIs of spontaneous preterm birth (sPTB) associated with exposure to total PM_2.5_ during pregnancy among subgroups stratified by the normalized difference vegetation index (NDVI) and tree canopy exposure in 200 m, 500 m, and 1000 m buffers **eFigure 6.** The adjusted odds ratios (ORs) with 95% CIs of spontaneous preterm birth (sPTB) associated with exposure to total PM_2.5_ during pregnancy among subgroups stratified by street view green space exposure in the 1000 m buffer (tertile groups) **eTable 1.** Pearson correlation coefficients between exposure to air pollutants throughout pregnancy for the study population **eTable 2.** Pearson correlation among different environmental exposures during pregnancy **eTable 3.** The adjusted odds ratios (ORs) with 95% CIs of sPTB associated with total PM_2.5_ and nonwildfire PM_2.5_ during pregnancy examined in the sensitivity analysis **eTable 4.** The adjusted odds ratios (ORs) with 95% CIs of sPTB associated with total PM_2.5_ and PM_2.5 _constituents during each trimester examined in all-trimester models **eTable 5.** The adjusted odds ratios (ORs) with 95% CIs of sPTB associated with PM_2.5_ constituents during pregnancy examined in copollutant models **eTable 6.** The adjusted odds ratios (ORs) and risk differences (RDs) with 95% CIs of sPTB associated with per-IQR increase in exposures to total PM_2.5_ and 5 PM_2.5_ constituents during pregnancy **eTable 7.** The results of associations between PM_2.5_ exposure during pregnancy and sPTB based on the propensity score matching
eReferences.

Supplement 2.
Data Sharing Statement
