# Supplementary material for: Fine Particulate Matter, Its Constituents, and Spontaneous Preterm Birth
Source: JAMA Netw Open. 2024 Nov 13;7(11):e2444593. doi: 10.1001/jamanetworkopen.2024.44593 (PMC11561696; doi:10.1001/jamanetworkopen.2024.44593)
Supplement: Supplement 1. — eMethods. eResults. eFigure 1. The composition of the Kaiser Permanente Southern California pregnancy cohort from 2008 to 2018 and the population selection process eFigure 2. The temporal trend of daily mean concentrations of total, nonwildfire, and wildfire-specific PM2.5 across California from 2007 to 2018 based on the census tract–level data eFigure 3. The mean exposure to total PM2.5, nonwildfire PM2.5, and wildfire-specific PM2.5 during pregnancy for the study population eFigure 4. The adjusted odds ratios (ORs) with 95% CIs of spontaneous preterm birth (sPTB) associated with exposure to total PM2.5 during pregnancy among subgroups stratified by street view green space exposure in 200 m and 500 m buffers eFigure 5. The adjusted odds ratios (ORs) with 95% CIs of spontaneous preterm birth (sPTB) associated with exposure to total PM2.5 during pregnancy among subgroups stratified by the normalized difference vegetation index (NDVI) and tree canopy exposure in 200 m, 500 m, and 1000 m buffers eFigure 6. The adjusted odds ratios (ORs) with 95% CIs of spontaneous preterm birth (sPTB) associated with exposure to total PM2.5 during pregnancy among subgroups stratified by street view green space exposure in the 1000 m buffer (tertile groups) eTable 1. Pearson correlation coefficients between exposure to air pollutants throughout pregnancy for the study population eTable 2. Pearson correlation among different environmental exposures during pregnancy eTable 3. The adjusted odds ratios (ORs) with 95% CIs of sPTB associated with total PM2.5 and nonwildfire PM2.5 during pregnancy examined in the sensitivity analysis eTable 4. The adjusted odds ratios (ORs) with 95% CIs of sPTB associated with total PM2.5 and PM2.5 constituents during each trimester examined in all-trimester models eTable 5. The adjusted odds ratios (ORs) with 95% CIs of sPTB associated with PM2.5 constituents during pregnancy examined in copollutant models eTable 6. The adjusted odds ratios (ORs) and risk dif [file jamanetwopen-e2444593-s001.pdf]

Supplemental Online Content

Jiao A, Reilly AN, Benmarhnia T, et al. Fine particulate matter, its constituents, and spontaneous preterm birth. *JAMA Netw Open*. 2024;7(11):e2444593. doi:10.1001/jamanetworkopen.2024.44593

eMethods.

eResults.

eFigure 1. The composition of the Kaiser Permanente Southern California pregnancy cohort from 2008 to 2018 and the population selection process

eFigure 2. The temporal trend of daily mean concentrations of total, nonwildfire, and wildfire-specific $PM_{2.5}$ across California from 2007 to 2018 based on the census tract–level data

eFigure 3. The mean exposure to total $PM_{2.5}$, nonwildfire $PM_{2.5}$, and wildfire-specific $PM_{2.5}$ during pregnancy for the study population

eFigure 4. The adjusted odds ratios (ORs) with 95% CIs of spontaneous preterm birth (sPTB) associated with exposure to total $PM_{2.5}$ during pregnancy among subgroups stratified by street view green space exposure in 200 m and 500 m buffers

eFigure 5. The adjusted odds ratios (ORs) with 95% CIs of spontaneous preterm birth (sPTB) associated with exposure to total $PM_{2.5}$ during pregnancy among subgroups stratified by the normalized difference vegetation index (NDVI) and tree canopy exposure in 200 m, 500 m, and 1000 m buffers

eFigure 6. The adjusted odds ratios (ORs) with 95% CIs of spontaneous preterm birth (sPTB) associated with exposure to total $PM_{2.5}$ during pregnancy among subgroups stratified by street view green space exposure in the 1000 m buffer (tertile groups)

eTable 1. Pearson correlation coefficients between exposure to air pollutants throughout pregnancy for the study population

eTable 2. Pearson correlation among different environmental exposures during pregnancy

eTable 3. The adjusted odds ratios (ORs) with 95% CIs of sPTB associated with total $PM_{2.5}$ and nonwildfire $PM_{2.5}$ during pregnancy examined in the sensitivity analysis

© 2024 Jiao A et al. *JAMA Network Open*.

eTable 4. The adjusted odds ratios (ORs) with 95% CIs of sPTB associated with total $PM_{2.5}$ and $PM_{2.5}$ constituents during each trimester examined in all-trimester models

eTable 5. The adjusted odds ratios (ORs) with 95% CIs of sPTB associated with $PM_{2.5}$ constituents during pregnancy examined in copollutant models

eTable 6. The adjusted odds ratios (ORs) and risk differences (RDs) with 95% CIs of sPTB associated with per-IQR increase in exposures to total $PM_{2.5}$ and 5 $PM_{2.5}$ constituents during pregnancy

eTable 7. The results of associations between $PM_{2.5}$ exposure during pregnancy and sPTB based on the propensity score matching

eReferences.

This supplemental material has been provided by the authors to give readers additional information about their work.

eMethods

Study Population

KPSC is a large integrated healthcare provider with 15 medical centers and 235 medical offices, serving approximately 21% of the Southern California population in 2020 [1]. The membership of KPSC has been reported to reflect the demographic and socioeconomic diversity of those living in this region [2-4]. There were 448,846 pregnancies from 341,341 individuals initially included in this cohort from 2008-2018. Pregnancies from individuals who were not KPSC members, with a gestational age of fewer than 20 weeks or larger than 47 weeks, without residential data, and resulting in multiple births or stillbirths were excluded from the cohort (eFigure 1). More details of this population have been described elsewhere [5,6].

Outcome Ascertainment

The gestational age was recorded in KPSC's electronic health records, ranging from 20 to 47 weeks (eFigure 1) [7]. For those who had early pregnancy ultrasound examinations, it was estimated mainly based on the first-trimester sonographic estimates [7,8]. For about 5% of pregnancies without sonographic estimates, it was estimated based on self-reported last menstrual period. Pregnancies less than 20 weeks of gestation were defined as miscarriages and excluded. Estimates based on self-reported last menstrual period may be inaccurate and result in extreme outliers. Hence, extremely post-term pregnancies with a gestational age of more than 47 weeks were further excluded from the cohort. A sensitivity analysis was conducted by restricting the study population to pregnancies with a gestational age between 20 and 43 weeks to avoid the potential impact of extreme outliers of gestational age.

Air Pollution Exposure Assessment

The ensemble model used to estimate daily total $PM_{2.5}$ concentrations incorporated multiple machine-learning algorithms (i.e., gradient boosting machine, random forest, and deep learning) with a large set of explanatory variables (e.g., satellite-derived aerosol properties, meteorological conditions, land-use variables, and thermal inversions) [9]. It showed a good performance with a prediction R^2 of 0.83 for all monitoring sites of the Environmental Protection Agency's Air Quality System in California.

In terms of the validated geoscience-derived model, it provided public data on $PM_{2.5}$ total mass and constituents based on ground-based measurements, satellite data, and chemical transport modeling [10,11]. The cross-validated annual performance was high for nitrate ($R^2 = 0.78$) and ammonium ($R^2 = 0.75$), followed by sulfate ($R^2 = 0.59$), organic matter ($R^2 = 0.52$), and black carbon ($R^2 = 0.42$) in the Southwestern United States [10,11].

For sensitivity analysis, we further obtained monthly data on nitrogen dioxide (NO_2), ozone (O_3-8h, the daily maximum from 10 AM to 6 PM), and $PM_{2.5}$ concentrations in California by implementing empirical Bayesian kriging (EBK) modeling relying on the ground-based measurements. The cross-validation R^2 of the EBK model was 0.74 for NO_2, 0.72 for O_3, and 0.65 for $PM_{2.5}$. Our previous publications have provided a detailed description of the EBK model [12,13].

Given the residential mobility, we excluded pregnancies if their residential information did not cover at least 75% of the entire pregnancy period (n = 20,802, 4.8%), similar to our previous studies [7]. Our previous publication has indicated that, compared to included pregnancies, there were higher percentages of excluded pregnancies from individuals of a younger age, more self-identified as Asian or Hispanic, with lower educational attainment or lower household income, or supported by Medicaid [7]. The electronic health records of KPSC documented the residential mobility during pregnancy for individuals who were members of KPSC. Individuals with a relatively lower socioeconomic status (e.g., a lower income level or with publicly funded insurance such as Medicaid) tend to be highly mobile and may find it hard to maintain a long-term KPSC membership, making it difficult to collect their full residential histories during pregnancy. For pregnancies whose missing residential data does not exceed 25% of the pregnancy period, we filled in the missing data using the address information from the later period for which data is available (n = 13,465, 3.1%). To check the robustness of our results, we estimated exposures and conducted the sensitivity analysis based on the original residential information of all pregnancies in the KPSC cohort (n = 429,839).

Green Space Exposure Assessment

For the street-view green space exposure assessment, we obtained street images from four main cardinal directions (i.e., 0°, 90°, 180°, and 270°) at each sampling point positioned at a 200-meter interval along the roadway. The validated deep learning model incorporated semantic segmentation to distinguish three types of vegetation: tree

© 2024 Jiao A et al. *JAMA Network Open.*

(e.g., canopy), low-lying vegetation (e.g., shrub, bush), and grass. The accuracy was high with a mean intersection over union (IoU) of 92.5%, indicating its effectiveness in actual image segmentation [14,15].

In sensitivity analysis, we applied additional buffers (i.e., 200 m and 500 m) and further examined green space exposure based on the Normalized Difference Vegetation Index (NDVI) and tree canopy coverage. The NDVI measurements were based on the Terra (MOD13Q1) satellite instrument of Moderate Resolution Imaging Spectroradiometer products from NASA with a spatial resolution of 250 m × 250 m and a temporal resolution of every 16 days [15]. Then, we obtained the percentage of tree canopy cover data at a 30 m resolution from the National Land Cover Database. We estimated the green space exposure for our study population mainly based on the NDVI and tree canopy data of the year 2013 (the mid-year of the study period), given that the annual green space coverage did not change substantially in our study region [16]. Detailed information on the green space exposure assessment has been provided in our previous publication [15].

Sensitivity analysis

Based on the main analyses for total $PM_{2.5}$ exposure during the entire pregnancy, we conducted the following sensitivity analyses to check the robustness of our findings: a) We performed models by further adjusting for different sets of confounders, including the average exposure to ambient temperature during pregnancy, insurance type, parity, smoking status, and some pre-gestational medical conditions (i.e., diabetes and chronic hypertension); b) We included only the first delivery of each individual in our cohort during the study period to avoid the potential impacts of any multiple pregnancies. From the entire cohort (n = 409,037), we included 304,570 (74.46%) pregnancies from 215,886 individuals with a single pregnancy and 88,684 individuals with multiple pregnancies, respectively; c) We restricted the study population to pregnancies with a gestational age between 20 and 43 weeks (n = 408,975, 99.98%); d) We conducted the analysis based on the original residential information before filling in missing data for all pregnancies (n = 429,839); e) We performed co-pollutant models by further adjusting for NO_2 and O_3; f) We applied the alternative exposure data of total $PM_{2.5}$ from the geoscience-derived model and the EBK model. All the analyses were also conducted for non-wildfire $PM_{2.5}$, except the analysis using exposure data from other sources.

For trimester-specific associations with total $PM_{2.5}$ and $PM_{2.5}$ constituents, we fitted a model by including all trimester-specific exposures in a single model (denoted as the all-trimester model) for each pollutant to reduce potential bias as a previous study suggested [6,17].

Next, for the associations with exposure to $PM_{2.5}$ constituents during the entire pregnancy, we co-adjusted five constituents to test the robustness of the single-pollutant model.

Furthermore, we examined the effect modification by street view green space, NDVI, and tree canopy exposure in different buffers (i.e., 200 m, 500 m, or 1000 m) in associations between total $PM_{2.5}$ and sPTB. To test the robustness of our findings, we also categorized the green space exposure into tertile groups (i.e., low, medium, and high exposure groups), another commonly used strategy in the analysis of effect modification [18-22].

To support the causal inference, we applied propensity score matching to examine the risk of sPTB associated with $PM_{2.5}$ exposure [23-25]. We dichotomized $PM_{2.5}$ exposure during pregnancy as high vs. low exposures based on the median level. A propensity score was derived to reflect the probability of a participant being exposed to a high level of $PM_{2.5}$ pollution during pregnancy given an observed set of characteristics. We calculated the propensity score using multivariate logistic regression and included the following variables in our main analysis, i.e., age, race and ethnicity, educational attainment, median household income, pre-pregnancy body mass index (BMI), season of conception, year of delivery, and county of residency. Participants in two exposure groups were matched 1:1 in random order on the propensity scores using a greedy algorithm and nearest-neighbor approach with a caliper of 0.1 using the PROC PSMATCH Procedure in SAS. The balance of characteristics between matched participants was assessed by standardized mean differences calculated using the R package "Tableone". After obtaining a matched dataset, we estimated the association between $PM_{2.5}$ exposure and sPTB based on logistic regression. In addition, to test the robustness of the analysis, we tried different matching strategies and included a different set of variables (including temperature exposure during pregnancy, insurance type, parity, smoking status, diabetes, and chronic hypertension).

© 2024 Jiao A et al. *JAMA Network Open.*

eResults

The exposure to total $PM_{2.5}$ and non-wildfire $PM_{2.5}$ during pregnancy measured based on the ensemble model were highly correlated with each other and both had low correlations with exposure to wildfire-specific $PM_{2.5}$ (eTable 1). They were moderately to highly correlated with exposure to $PM_{2.5}$ constituents ($0.36 \leq r \leq 0.71$) estimated based on the geoscience-derived model, with the lowest correlation with $PM_{2.5}$ sulfate and the highest correlation with $PM_{2.5}$ nitrate. Wildfire-specific $PM_{2.5}$ had low correlations with all five $PM_{2.5}$ constituents ($-0.15 \leq r \leq 0.26$).

The total green space exposure showed a high correlation with trees (r = 0.83) but low correlations with low-lying vegetation (r = 0.21) and grass (r = 0.17) (eTable 2). The exposure to total $PM_{2.5}$ and non-wildfire $PM_{2.5}$ during pregnancy had low correlations with total and specific green space exposure, except moderate and negative correlations with exposure to low-lying vegetation. There were little correlations between exposure to wildfire-specific $PM_{2.5}$ and green space. In addition, we observed little correlation between temperature exposure and other environmental exposures.

eFigure 2 depicts the temporal trend of daily average concentrations of total, non-wildfire, and wildfire-specific $PM_{2.5}$ across California from 2007 to 2018. The peaks in total and wildfire-specific $PM_{2.5}$ concentrations were both observed in the years 2008 and 2018. eFigure 3 displays the average exposure to total, non-wildfire, and wildfire-specific $PM_{2.5}$ of the study population during pregnancy. The maximum values of exposure to total, non-wildfire, and wildfire-specific $PM_{2.5}$ were 24.65 $\mu g/m^3$, 24.49 $\mu g/m^3$, and 3.99 $\mu g/m^3$, respectively.

The ORs of iPTB associated with per IQR increase in total $PM_{2.5}$ exposure were 0.81 (95% CI, 0.79-0.83; $P < .001$), 0.87 (95% CI, 0.85-0.89; $P < .001$), 0.87 (95% CI, 0.85-0.89; $P < .001$), and 0.87 (95% CI, 0.86-0.89; $P < .001$) during the entire pregnancy, first, second, and third trimester, respectively.

We observed similar but lower associations from the unadjusted models compared to the adjusted models in the main analysis (eTable 3). For the sensitivity analysis of associations with total $PM_{2.5}$ and non-wildfire $PM_{2.5}$ exposure during the entire pregnancy, (a) Models further adjusted for different sets of covariates changed the results minimally; (b) We observed similar results before vs. after controlling for multiple deliveries; (c) Restricting the study population to pregnancies with a gestational age of 20-43 weeks did not change the observed associations; (d) The results without filling in missing residential information were similar to those of our main analysis; (e) Co-adjustment of NO_2 and O_3 did not change our conclusions; (f) By using total $PM_{2.5}$ exposure data estimated based on the geoscience-derived model and the EBK model, we observed slightly attenuated associations compared to our main analysis, while our conclusion remained unchanged. The results of non-wildfire $PM_{2.5}$ exposure were very close to those of the total $PM_{2.5}$.

The all-trimester models indicated that the second trimester might be the most susceptible exposure window, consistent with our main analysis (eTable 4).

The results derived from co-pollutant models fitted for five $PM_{2.5}$ constituents did not change substantially compared to those of the single-pollutant models (eTable 5). We observed that the association with $PM_{2.5}$ nitrate was increased after adjusting for $PM_{2.5}$ ammonium, while the association with $PM_{2.5}$ ammonium was changed to be negative. This is likely due to the high correlation between those two constituents (r = 0.8). In addition, we found the associations with the other four constituents were slightly attenuated after adjusting for $PM_{2.5}$ black carbon.

We examined the effect modification by street view-based green space, NDVI, and tree canopy exposure in different buffers (e.g., 200 m, 500 m, or 1000 m) around the residential area (eFigures 4 and 5). We found similar results indicating that individuals living in areas with more exposure to total green space or trees tended to have a lower risk of sPTB associated with exposure to total $PM_{2.5}$. The results regarding effect modification by street-view green space based on tertile groups were similar to the results in the main analysis, indicating that a high level of exposure to total green space and trees can attenuate the association between $PM_{2.5}$ and exposure, while exposures to low-lying vegetation and grass modified the association in another direction (eFigure 6).

We reported the risk differences (RDs) for exposures to total $PM_{2.5}$ and five $PM_{2.5}$ constituents during pregnancy. Every interquartile range (IQR) increase in exposures to $PM_{2.5}$, sulfate, nitrate, ammonium, organic matter, and black carbon was associated with increases in odds of spontaneous preterm birth (sPTB) by 0.74%, 0.27%, 0.49%, 0.18%, 0.27%, and 0.68%, respectively (eTable 6).

By applying the propensity score matching, we found that higher exposure to $PM_{2.5}$ during pregnancy was associated with a 7% increased odds of sPTB, which supports our findings in the main analysis (eTable 7). The

results based on different matching strategies were consistent and indicated significantly positive associations between $PM_{2.5}$ and sPTB.

© 2024 Jiao A et al. *JAMA Network Open*.

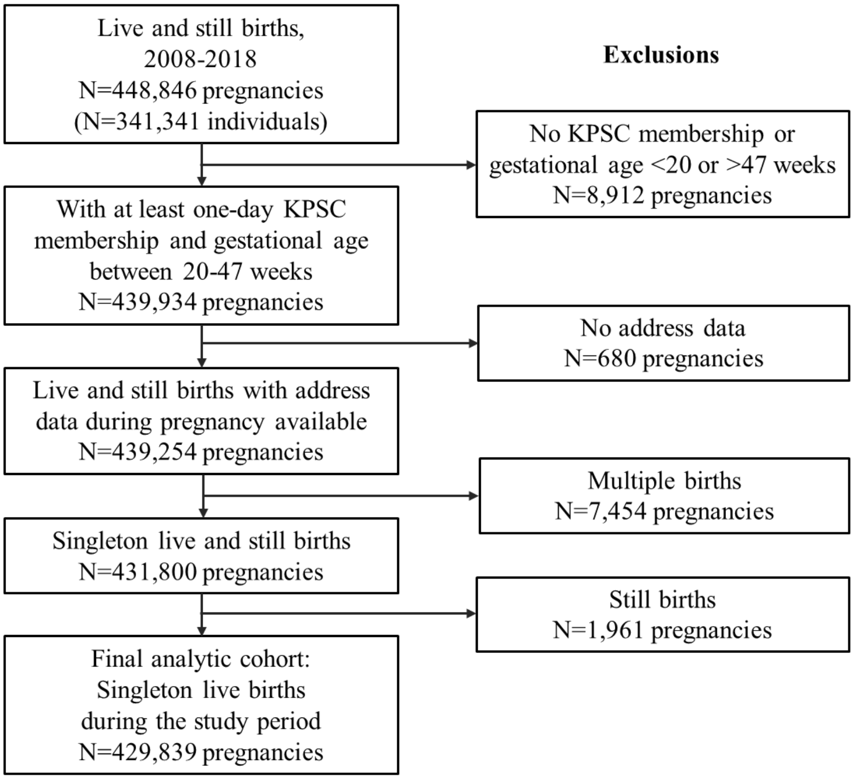

eFigure 1. The composition of the Kaiser Permanente Southern California pregnancy cohort from 2008 to 2018 and the population selection process.

© 2024 Jiao A et al. *JAMA Network Open*.

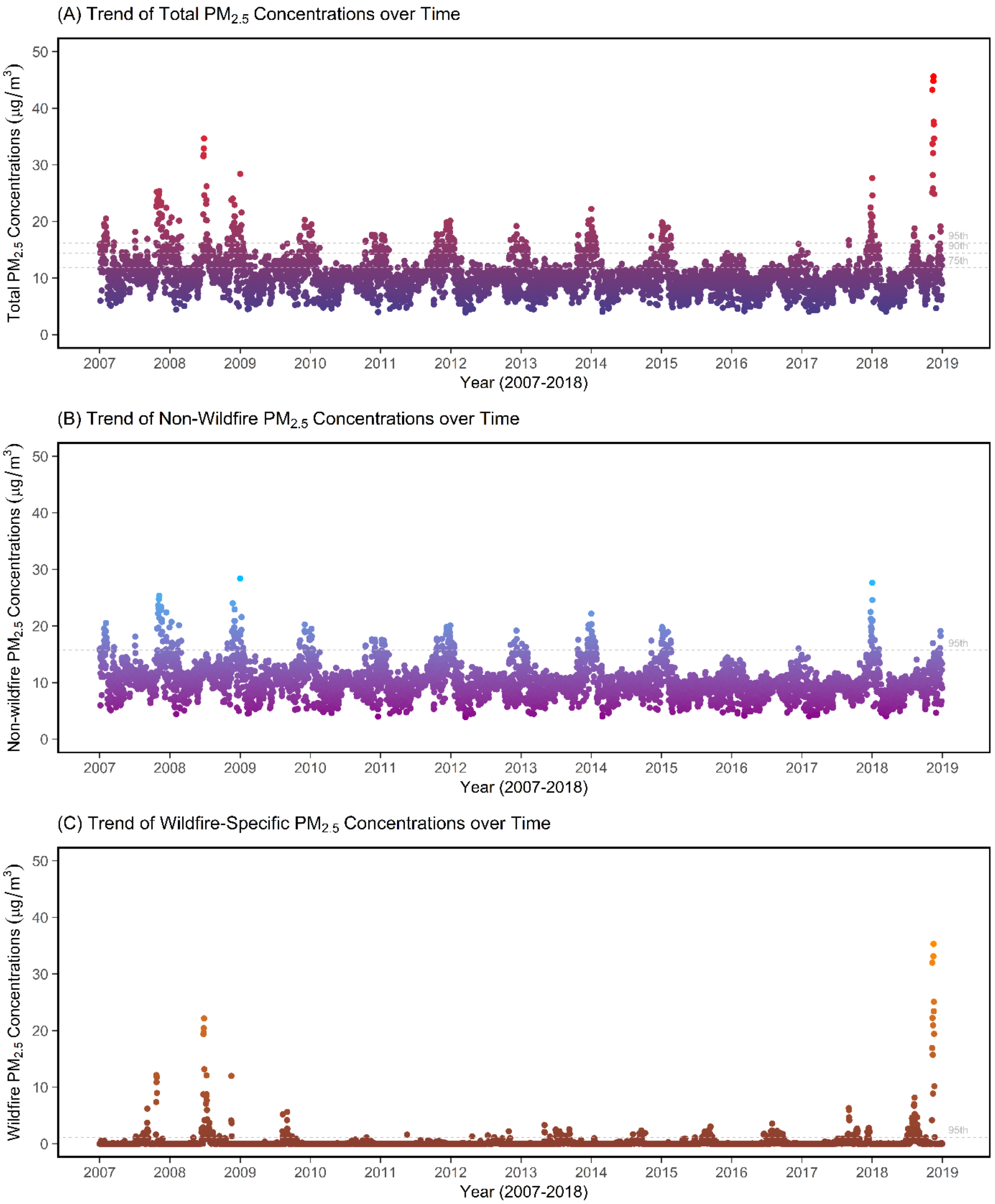

eFigure 2. The temporal trend of daily average concentrations of total, non-wildfire, and wildfire-specific $PM_{2.5}$ across California from 2007 to 2018 based on the census tract level data. The 75th, 90th, and 95th percentiles of the total $PM_{2.5}$ were 11.83, 14.43, and 16.19 μg/m^3, respectively. The 95th percentiles of non-wildfire and wildfire-specific $PM_{2.5}$ were 15.72 and 1.17 μg/m^3, respectively.

© 2024 Jiao A et al. *JAMA Network Open*.

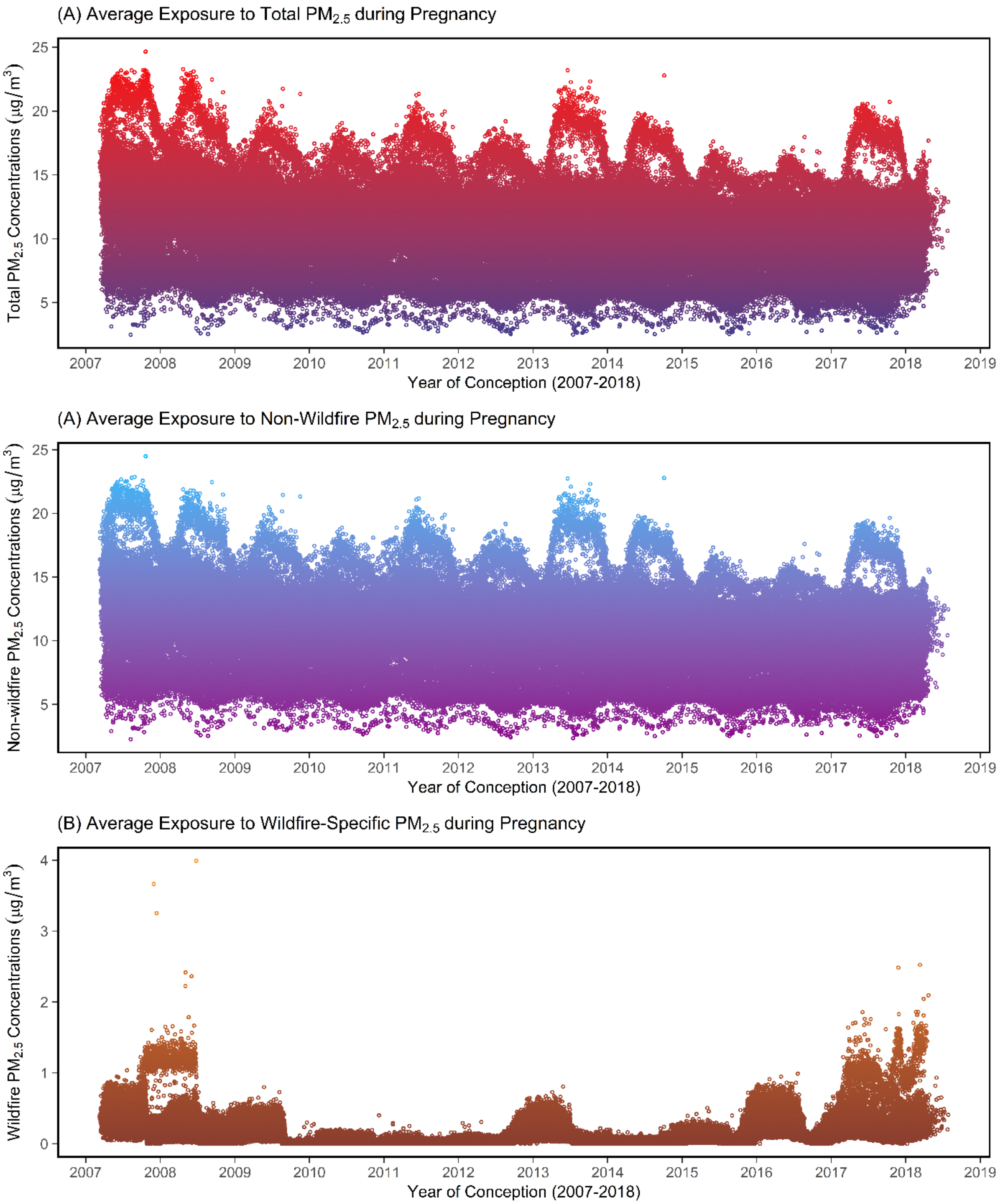

eFigure 3. The average exposure to total $PM_{2.5}$, non-wildfire $PM_{2.5}$, and wildfire-specific $PM_{2.5}$ during pregnancy for the study population.

© 2024 Jiao A et al. *JAMA Network Open*.

Subgroup	OR (95% CI)	P Value
Street View Green Space in 200 m		
Total Green Space		.08
<50th	1.17 (1.13-1.20)	
>=50th	1.13 (1.09-1.16)	
Trees		.07
<50th	1.16 (1.13-1.20)	
>=50th	1.12 (1.08-1.16)	
Low-lying Vegetation		.18
<50th	1.12 (1.08-1.16)	
>=50th	1.15 (1.12-1.19)	
Grass		.26
<50th	1.13 (1.09-1.17)	
>=50th	1.16 (1.12-1.19)	
Street View Green Space in 500 m		
Total Green Space		.09
<50th	1.17 (1.13-1.21)	
>=50th	1.13 (1.10-1.16)	
Trees		.01
<50th	1.17 (1.14-1.20)	
>=50th	1.11 (1.07-1.15)	
Low-lying Vegetation		.02
<50th	1.10 (1.06-1.14)	
>=50th	1.16 (1.13-1.19)	
Grass		.55
<50th	1.14 (1.10-1.18)	
>=50th	1.15 (1.12-1.18)	

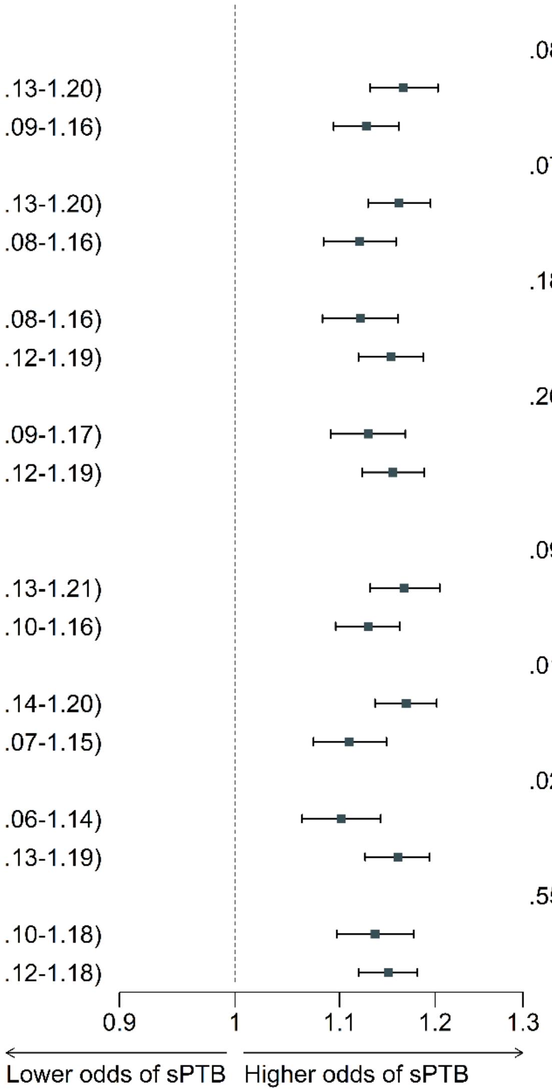

eFigure 4. The adjusted odds ratios (ORs) with 95% confidence intervals (CIs) of spontaneous preterm birth (sPTB) associated with exposure to total $PM_{2.5}$ during pregnancy among subgroups stratified by street-view green space exposure in 200 m and 500 m buffers. Models adjusted for age, race and ethnicity, educational attainment, median household income, pre-pregnancy body mass index, season of conception, year of delivery, county of residence, and the respective effect modifier. The *P* value is for the interaction term between $PM_{2.5}$ exposure and each effect modifier.

© 2024 Jiao A et al. *JAMA Network Open*.

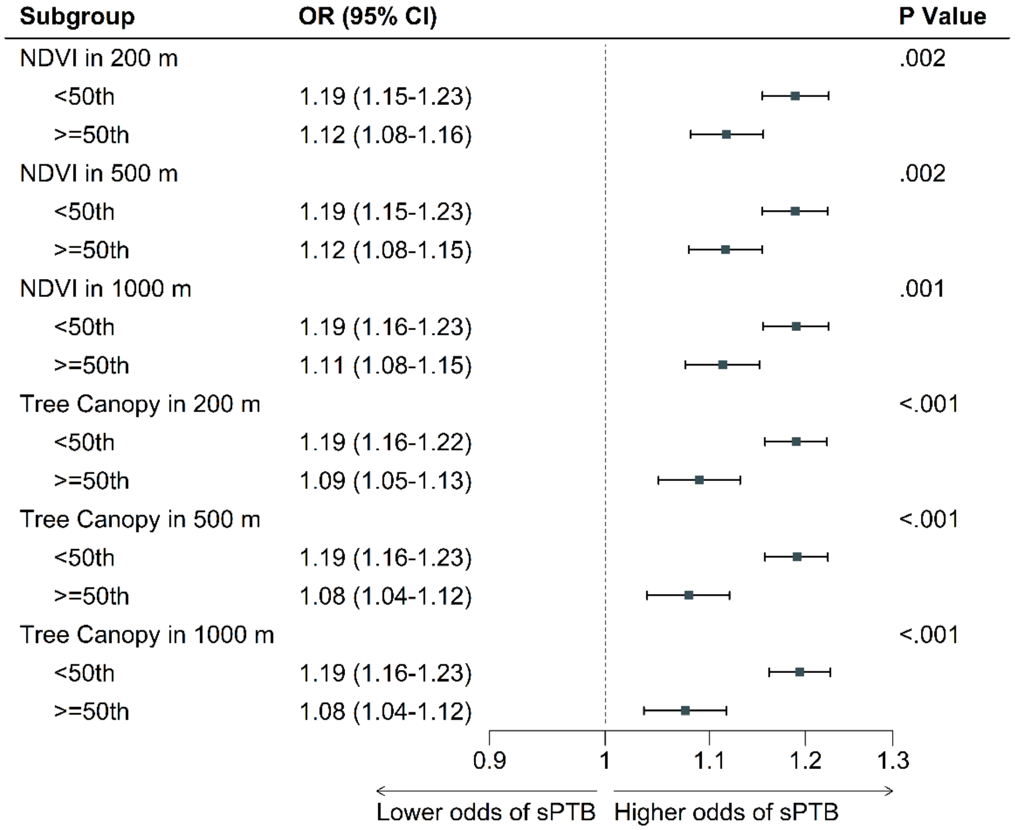

eFigure 5. The adjusted odds ratios (ORs) with 95% confidence intervals (CIs) of spontaneous preterm birth (sPTB) associated with exposure to total $PM_{2.5}$ during pregnancy among subgroups stratified by the Normalized Difference Vegetation Index (NDVI) and tree canopy exposure in 200 m, 500 m, and 1000 m buffers. Models adjusted for age, race and ethnicity, educational attainment, median household income, pre-pregnancy body mass index, season of conception, year of delivery, county of residence, and the respective effect modifier. The *P* value is for the interaction term between $PM_{2.5}$ exposure and each effect modifier.

© 2024 Jiao A et al. *JAMA Network Open*.

Subgroup	OR (95% CI)	P Value
Green Space Exposure		
Total Green Space		.001
Low (1st tertile)	1.18 (1.13-1.22)	
Medium (2nd tertile)	1.19 (1.15-1.23)	
High (3rd tertile)	1.10 (1.06-1.14)	
Trees		.002
Low (1st tertile)	1.17 (1.14-1.21)	
Medium (2nd tertile)	1.16 (1.11-1.21)	
High (3rd tertile)	1.08 (1.04-1.13)	
Low-lying Vegetation		<.001
Low (1st tertile)	1.05 (1.01-1.10)	
Medium (2nd tertile)	1.16 (1.12-1.21)	
High (3rd tertile)	1.17 (1.13-1.21)	
Grass		.02
Low (1st tertile)	1.11 (1.06-1.16)	
Medium (2nd tertile)	1.12 (1.07-1.16)	
High (3rd tertile)	1.18 (1.14-1.21)	
Wildfire Smoke Exposure		<.001
Low (1st tertile)	1.08 (1.04-1.12)	
Medium (2nd tertile)	1.23 (1.18-1.28)	
High (3rd tertile)	1.20 (1.16-1.24)	

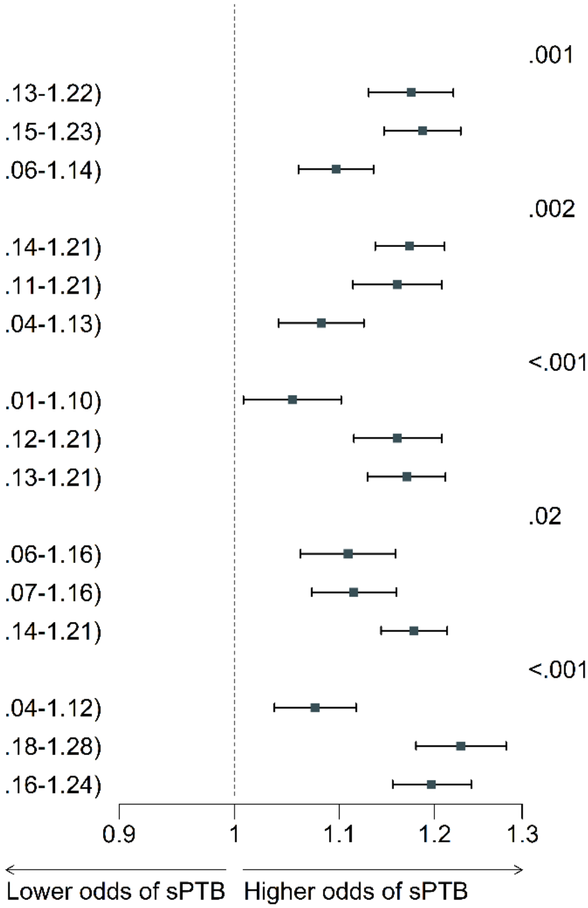

eFigure 6. The adjusted odds ratios (ORs) with 95% confidence intervals (CIs) of spontaneous preterm birth (sPTB) associated with exposure to total $PM_{2.5}$ during pregnancy among subgroups stratified by street-view green space exposure in the 1000 m buffer (tertile groups). Models adjusted for age, race and ethnicity, educational attainment, median household income, pre-pregnancy body mass index, season of conception, year of delivery, county of residence, and the respective effect modifier. The *P* value is for the interaction term between $PM_{2.5}$ exposure and each effect modifier.

© 2024 Jiao A et al. *JAMA Network Open*.

eTable 1. Pearson correlation coefficients between exposure to air pollutants throughout pregnancy for the study population.

	Total $PM_{2.5}$ [a]	Non-wildfire $PM_{2.5}$ [a]	Wildfire-specific $PM_{2.5}$ [a]	$PM_{2.5}$ sulfate	$PM_{2.5}$ nitrate	$PM_{2.5}$ ammonium	$PM_{2.5}$ organic matter
Non-wildfire $PM_{2.5}$	0.99 [c]	-	-	-	-	-	-
Wildfire-specific $PM_{2.5}$	0.23 [c]	0.17 [c]	-	-	-	-	-
$PM_{2.5}$ sulfate [b]	0.37 [c]	0.36 [c]	0.20 [c]	-	-	-	-
$PM_{2.5}$ nitrate [b]	0.71 [c]	0.71 [c]	0.12 [c]	0.33 [c]	-	-	-
$PM_{2.5}$ ammonium [b]	0.61 [c]	0.61 [c]	0.13 [c]	0.44 [c]	0.80 [c]	-	-
$PM_{2.5}$ organic matter [b]	0.70 [c]	0.69 [c]	0.26 [c]	0.31 [c]	0.66 [c]	0.55 [c]	-
$PM_{2.5}$ black carbon [b]	0.57 [c]	0.59 [c]	-0.15 [c]	0.22 [c]	0.54 [c]	0.52 [c]	0.72 [c]

[a] The $PM_{2.5}$ data were obtained from the ensemble model that incorporated multiple machine-learning algorithms at the census tract level.
[b] The $PM_{2.5}$ constituents data were from the geoscience-derived model at a spatial resolution of 1 km.
[c] The correlation with a *P* value <.001.

© 2024 Jiao A et al. *JAMA Network Open*.

eTable 2. Pearson correlation among different environmental exposures during pregnancy.

	Total $PM_{2.5}$ [a]	Non-wildfire $PM_{2.5}$ [a]	Wildfire-specific $PM_{2.5}$ [a]	Total green space	Trees	Low-lying vegetation	Grass
Non-wildfire $PM_{2.5}$	0.99 [d]	-	-	-	-	-	-
Wildfire-specific $PM_{2.5}$	0.23 [d]	0.17 [d]	-	-	-	-	-
Total green space [b]	-0.16 [d]	-0.16 [d]	-0.03 [d]	-	-	-	-
Trees [b]	0.08 [d]	0.08 [d]	-0.02 [d]	0.83 [d]	-	-	-
Low-lying vegetation [b]	-0.49 [d]	-0.50 [d]	-0.05 [d]	0.21 [d]	-0.25 [d]	-	-
Grass [b]	-0.16 [d]	-0.17 [d]	0.04 [d]	0.17 [d]	-0.29 [d]	0.27 [d]	-
Temperature [c]	0.05 [d]	0.04 [d]	0.14 [d]	0.02 [d]	0.01 [d]	-0.09 [d]	0.11 [d]

[a] The $PM_{2.5}$ data were obtained from the ensemble model that incorporated multiple machine-learning algorithms at the census tract level.
[b] The green space data were estimated based on street view images.
[c] The temperature exposure was estimated based on daily maximum temperature data.
[d] The correlation with a *P* value <.001.

© 2024 Jiao A et al. *JAMA Network Open*.

eTable 3. The adjusted odds ratios (ORs) with 95% confidence intervals (CIs) of sPTB associated with total $PM_{2.5}$ and non-wildfire $PM_{2.5}$ during pregnancy examined in the sensitivity analysis.

	Total $PM_{2.5}$	Non-wildfire $PM_{2.5}$
Main model [a]	1.15 (1.12-1.18)	1.15 (1.13-1.18)
Unadjusted model [b]	1.09 (1.07-1.10)	1.09 (1.07-1.11)
Model with more covariates		
Model 1 [c]	1.15 (1.12-1.18)	1.15 (1.13-1.18)
Model 2 [d]	1.15 (1.12-1.17)	1.15 (1.12-1.18)
Model 3 [e]	1.15 (1.12-1.17)	1.15 (1.12-1.18)
Model accounting for multiple deliveries [f]	1.15 (1.12-1.18)	1.15 (1.12-1.19)
Model with restricted gestational age <=43 [g]	1.15 (1.12-1.18)	1.15 (1.13-1.18)
Model before filling in missing residential data [h]	1.14 (1.12-1.17)	1.15 (1.12-1.18)
Co-pollutant model [i]		
+ NO_2	1.15 (1.12-1.19)	1.16 (1.13-1.19)
+ O_3	1.16 (1.13-1.18)	1.16 (1.13-1.19)
Model using $PM_{2.5}$ exposure from other data sources		
Geoscience model [j]	1.11 (1.07-1.14)	-
Kriging model [k]	1.12 (1.09-1.16)	-

Abbreviations: NO_2, nitrogen dioxide; O_3, ozone; $PM_{2.5}$, particulate matter less than or equal to 2.5 μm; sPTB, spontaneous preterm birth.

[a] Main model: model in the main analysis adjusted for age, race and ethnicity, educational attainment, median household income, pre-pregnancy body mass index, season of conception, year of delivery, and county of residence.

[b] Unadjusted model: the model including only the exposure variable.

[c] Model 1: the main model further adjusted for average exposure to temperature during pregnancy.

[d] Model 2: the main model further adjusted for average exposure to temperature during pregnancy, insurance type, parity, and smoking status.

[e] Model 3: the main model further adjusted for average exposure to temperature during pregnancy, insurance type, parity, smoking status, pre-existing diabetes, and chronic hypertension.

[f] The model including only the first delivery for each participant during the study period.

[g] The model excluding extremely post-term pregnancies > 43 weeks of gestation.

[h] The model with exposure data before filling in missing residential information.

[i] The main model was further adjusted for average exposure to NO_2 and O_3 during the entire pregnancy, respectively.

[j] The $PM_{2.5}$ exposure data were obtained from the geoscience-derived model.

[k] The $PM_{2.5}$ exposure data were obtained from the empirical Bayesian Kriging model.

© 2024 Jiao A et al. *JAMA Network Open.*

eTable 4. The adjusted odds ratios (ORs) with 95% confidence intervals (CIs) of sPTB associated with total $PM_{2.5}$ and $PM_{2.5}$ constituents during each trimester examined in all-trimester models.

	Exposure window [a]		
	First trimester	Second trimester	Third trimester
Total $PM_{2.5}$	1.03 (1.01-1.05)	1.06 (1.04-1.08)	1.05 (1.03-1.07)
$PM_{2.5}$ sulfate	1.03 (1.01-1.05)	1.01 (1.00-1.03)	1.02 (1.01-1.04)
$PM_{2.5}$ nitrate	1.02 (1.00-1.04)	1.05 (1.02-1.07)	1.02 (1.00-1.04)
$PM_{2.5}$ ammonium	1.01 (0.99-1.03)	1.03 (1.00-1.05)	1.00 (0.98-1.02)
$PM_{2.5}$ organic matter	1.00 (0.97-1.02)	1.06 (1.03-1.09)	1.00 (0.98-1.03)
$PM_{2.5}$ black carbon	1.04 (1.00-1.07)	1.12 (1.08-1.17)	0.99 (0.96-1.03)

Abbreviations: $PM_{2.5}$, particulate matter less than or equal to 2.5 μm; sPTB, spontaneous preterm birth.
[a] Models adjusted for age, race and ethnicity, educational attainment, median household income, pre-pregnancy body mass index, season of conception, year of delivery, and county of residence.

© 2024 Jiao A et al. *JAMA Network Open*.

eTable 5. The adjusted odds ratios (ORs) with 95% confidence intervals (CIs) of sPTB associated with $PM_{2.5}$ constituents during pregnancy examined in co-pollutant models.

	Single pollutant [a]	Co-pollutant [a]				
		+ $PM_{2.5}$ sulfate	+ $PM_{2.5}$ nitrate	+ $PM_{2.5}$ ammonium	+ $PM_{2.5}$ organic matter	+ $PM_{2.5}$ black carbon
$PM_{2.5}$ sulfate	1.06 (1.03-1.09)	-	1.03 (1.01-1.06)	1.06 (1.03-1.09)	1.05 (1.02-1.08)	1.03 (1.00-1.06)
$PM_{2.5}$ nitrate	1.09 (1.06-1.13)	1.08 (1.05-1.11)	-	1.19 (1.13-1.26)	1.09 (1.05-1.13)	1.04 (1.00-1.08)
$PM_{2.5}$ ammonium	1.03 (1.00-1.06)	1.00 (0.97-1.04)	0.90 (0.85-0.94)	-	1.01 (0.98-1.04)	0.98 (0.95-1.01)
$PM_{2.5}$ organic matter	1.05 (1.02-1.08)	1.04 (1.01-1.07)	1.00 (0.97-1.04)	1.05 (1.02-1.08)	-	0.96 (0.92-1.00)
$PM_{2.5}$ black carbon	1.15 (1.11-1.20)	1.14 (1.09-1.19)	1.12 (1.07-1.18)	1.17 (1.12-1.22)	1.20 (1.14-1.27)	-

Abbreviations: $PM_{2.5}$, particulate matter less than or equal to 2.5 μm; sPTB, spontaneous preterm birth.

[a] Models adjusted for age, race and ethnicity, educational attainment, median household income, pre-pregnancy body mass index, season of conception, year of delivery, and county of residence.

© 2024 Jiao A et al. *JAMA Network Open*.

eTable 6. The adjusted odds ratios (ORs) and risk differences (RDs) with 95% confidence intervals (CIs) of sPTB associated with per interquartile range (IQR) increase in exposures to total $PM_{2.5}$ and five $PM_{2.5}$ constituents during pregnancy.

	ORs (95% CI) [a]	RDs (95% CI) [a]
Total $PM_{2.5}$	1.16 (1.14-1.19)	0.74% (0.64%-0.84%)
$PM_{2.5}$ sulfate	1.07 (1.04-1.11)	0.27% (0.13%-0.41%)
$PM_{2.5}$ nitrate	1.10 (1.07-1.14)	0.49% (0.33%-0.64%)
$PM_{2.5}$ ammonium	1.03 (1.00-1.07)	0.18% (0.04%-0.33%)
$PM_{2.5}$ organic matter	1.06 (1.03-1.09)	0.27% (0.14%-0.41%)
$PM_{2.5}$ black carbon	1.15 (1.11-1.21)	0.68% (0.48%-0.88%)

Abbreviations: $PM_{2.5}$, particulate matter less than or equal to 2.5 μm; sPTB, spontaneous preterm birth.
[a] Models adjusted for age, race and ethnicity, educational attainment, median household income, pre-pregnancy body mass index, season of conception, year of delivery, and county of residence. Since the model with a random effect of residence county cannot converge when calculating risk differences using an identity link, we included the county as a fixed effect in these models. The results were comparable with our main analysis where the county was included as a random effect.

© 2024 Jiao A et al. *JAMA Network Open*.

eTable 7. The results of associations between $PM_{2.5}$ exposure during pregnancy and sPTB based on the propensity score matching.

	Model 1 [a]	Model 2 [b]	Model 3 [c]	Model 4 [d]
Matched pairs	101,679	103,899	105,811	98,890
Standardized mean difference				
Propensity Score	0.073	0.100	0.150	0.076
Age	0.078	0.090	0.061	0.011
Race and ethnicity	0.087	0.122	0.088	0.004
Education level	0.037	0.024	0.044	0.022
Household income	0.008	0.012	0.011	0.018
Pre-pregnancy BMI	0.046	0.047	0.051	0.003
Season of conception	0.011	0.012	0.019	0.014
Year of delivery	0.179	0.179	0.237	0.146
County	0.241	0.226	0.261	0.141
Temperature exposure	-	-	-	0.023
Insurance type	-	-	-	0.003
Parity	-	-	-	0.003
Smoking status	-	-	-	0.012
Diabetes	-	-	-	0.003
Chronic hypertension	-	-	-	0.001
Univariate regression model [e]	1.07 (1.03-1.11)	1.09 (1.04-1.13)	1.07 (1.03-1.12)	1.05 (1.01-1.10)
Multivariate regression model [e]	1.07 (1.02-1.11)	1.08 (1.04-1.13)	1.08 (1.03-1.12)	1.06 (1.02-1.11)

Abbreviations: $PM_{2.5}$, particulate matter less than or equal to 2.5 µm; sPTB, spontaneous preterm birth.
[a] Main model, adjusted for age, race and ethnicity, educational attainment, median household income, pre-pregnancy body mass index, season of conception, year of delivery, and county of residence.
[b] The model was adjusted for age, race and ethnicity, educational attainment, median household income, pre-pregnancy body mass index, season of conception, year of delivery, and county of residence. The logit of the propensity score was used in computing differences between pairs of observations.
[c] The model was adjusted for age, race and ethnicity, educational attainment, median household income, pre-pregnancy body mass index, season of conception, year of delivery, and county of residence. The caliper was 0.2.
[d] The model was adjusted for age, race and ethnicity, educational attainment, median household income, pre-pregnancy body mass index, season of conception, year of delivery, county of residence, temperature, insurance type, parity, smoking status, diabetes, and chronic hypertension.
[e] The results were represented by odds ratios with 95% confidence intervals.

© 2024 Jiao A et al. *JAMA Network Open.*

eReferences

1. Chen W, Xie F, McCarthy DP, et al. Research data warehouse: using electronic health records to conduct population-based observational studies. *JAMIA Open*. Jul 2023;6(2):ooad039. doi:10.1093/jamiaopen/ooad039
2. Koebnick C, Langer-Gould AM, Gould MK, et al. Sociodemographic characteristics of members of a large, integrated health care system: comparison with US Census Bureau data. *The Permanente journal*. Summer 2012;16(3):37-41. doi:10.7812/TPP/12-031
3. Mefford MT, Zhuang Z, Liang Z, et al. Temporal trends in heart failure mortality in an integrated healthcare delivery system, California, and the US, 2001-2017. *BMC Cardiovasc Disord*. May 26 2021;21(1):261. doi:10.1186/s12872-021-02075-6
4. Chen W, Yao J, Liang Z, et al. Temporal Trends in Mortality Rates among Kaiser Permanente Southern California Health Plan Enrollees, 2001-2016. *The Permanente journal*. 2019;23doi:10.7812/TPP/18-213
5. Sun Y, Li X, Benmarhnia T, et al. Exposure to air pollutant mixture and gestational diabetes mellitus in Southern California: Results from electronic health record data of a large pregnancy cohort. *Environ Int*. Sep 24 2021;158:106888. doi:10.1016/j.envint.2021.106888
6. Jiao A, Sun Y, Avila C, et al. Maternal exposure to ambient air pollution mixture and premature rupture of membranes: Evidence from a large cohort in Southern California (2008-2018). *Environ Int*. Jul 2023;177:108030. doi:10.1016/j.envint.2023.108030
7. Jiao A, Sun Y, Avila C, et al. Analysis of Heat Exposure During Pregnancy and Severe Maternal Morbidity. *JAMA network open*. Sep 5 2023;6(9):e2332780. doi:10.1001/jamanetworkopen.2023.32780
8. Sun Y, Headon KS, Jiao A, et al. Association of Antepartum and Postpartum Air Pollution Exposure With Postpartum Depression in Southern California. *JAMA network open*. Oct 2 2023;6(10):e2338315. doi:10.1001/jamanetworkopen.2023.38315
9. Aguilera R, Luo N, Basu R, et al. A novel ensemble-based statistical approach to estimate daily wildfire-specific PM(2.5) in California (2006-2020). *Environ Int*. Jan 2023;171:107719. doi:10.1016/j.envint.2022.107719
10. Meng J, Li C, Martin RV, van Donkelaar A, Hystad P, Brauer M. Estimated Long-Term (1981-2016) Concentrations of Ambient Fine Particulate Matter across North America from Chemical Transport Modeling, Satellite Remote Sensing, and Ground-Based Measurements. *Environ Sci Technol*. May 7 2019;53(9):5071-5079. doi:10.1021/acs.est.8b06875
11. van Donkelaar A, Martin RV, Li C, Burnett RT. Regional Estimates of Chemical Composition of Fine Particulate Matter Using a Combined Geoscience-Statistical Method with Information from Satellites, Models, and Monitors. *Environ Sci Technol*. Mar 5 2019;53(5):2595-2611. doi:10.1021/acs.est.8b06392
12. Wu J, Laurent O, Li L, Hu J, Kleeman M. Adverse reproductive health outcomes and exposure to gaseous and particulate-matter air pollution in pregnant women. *Research Reports: Health Effects Institute*. 2016;2016
13. Laurent O, Hu J, Li L, et al. A Statewide Nested Case-Control Study of Preterm Birth and Air Pollution by Source and Composition: California, 2001-2008. *Environ Health Perspect*. Sep 2016;124(9):1479-86. doi:10.1289/ehp.1510133
14. Sun Y, Wang X, Zhu J, et al. Using machine learning to examine street green space types at a high spatial resolution: Application in Los Angeles County on socioeconomic disparities in exposure. *Science of The Total Environment*. 2021;787:147653. doi:10.1016/j.scitotenv.2021.147653
15. Sun Y, Molitor J, Benmarhnia T, et al. Association between urban green space and postpartum depression, and the role of physical activity: a retrospective cohort study in Southern California. *The Lancet Regional Health - Americas*. 2023:100462. doi:10.1016/j.lana.2023.100462
16. Sun Y, Sheridan P, Laurent O, et al. Associations between green space and preterm birth: Windows of susceptibility and interaction with air pollution. *Environ Int*. Sep 2020;142:105804. doi:10.1016/j.envint.2020.105804
17. Wilson A, Chiu YM, Hsu HL, Wright RO, Wright RJ, Coull BA. Potential for Bias When Estimating Critical Windows for Air Pollution in Children's Health. *Am J Epidemiol*. Dec 1 2017;186(11):1281-1289. doi:10.1093/aje/kwx184
18. Choi HM, Lee W, Roye D, et al. Effect modification of greenness on the association between heat and mortality: A multi-city multi-country study. *EBioMedicine*. Oct 2022;84:104251. doi:10.1016/j.ebiom.2022.104251
19. Song J, Lu Y, Zhao Q, et al. Effect modifications of green space and blue space on heat-mortality association in Hong Kong, 2008-2017. *Sci Total Environ*. Sep 10 2022;838(Pt 2):156127. doi:10.1016/j.scitotenv.2022.156127

20. Miron-Celis M, Talarico R, Villeneuve PJ, et al. Critical windows of exposure to air pollution and gestational diabetes: assessing effect modification by maternal pre-existing conditions and environmental factors. *Environ Health*. Mar 15 2023;22(1):26. doi:10.1186/s12940-023-00974-z
21. Son JY, Sabath MB, Lane KJ, et al. Long-term Exposure to PM2.5 and Mortality for the Older Population: Effect Modification by Residential Greenness. *Epidemiology*. Jul 1 2021;32(4):477-486. doi:10.1097/EDE.0000000000001348
22. Poulsen AH, Sorensen M, Hvidtfeldt UA, et al. Air pollution and myocardial infarction; effect modification by sociodemographic and environmental factors. A cohort study from Denmark. *Environ Res*. Jul 15 2023;229:115905. doi:10.1016/j.envres.2023.115905
23. Ebrahim Valojerdi A, Janani L. A brief guide to propensity score analysis. *Med J Islam Repub Iran*. 2018;32:122. doi:10.14196/mjiri.32.122
24. Grool AM, Aglipay M, Momoli F, et al. Association Between Early Participation in Physical Activity Following Acute Concussion and Persistent Postconcussive Symptoms in Children and Adolescents. *JAMA*. Dec 20 2016;316(23):2504-2514. doi:10.1001/jama.2016.17396
25. Qin MM, Khoshnevis N, Dominici F, Braun D, Zanobetti A, Mork D. Comparing traditional and causal inference methodologies for evaluating impacts of long-term air pollution exposure on hospitalization with Alzheimer's disease and related dementias. *Am J Epidemiol*. Jun 21 2024;doi:10.1093/aje/kwae133

© 2024 Jiao A et al. *JAMA Network Open*.
