# Supplementary material for: Fine Particulate Matter, Its Constituents, and Spontaneous Preterm Birth
Source: JAMA Netw Open. 2024 Nov 13;7(11):e2444593. doi: 10.1001/jamanetworkopen.2024.44593 (PMC11561696; doi:10.1001/jamanetworkopen.2024.44593)
Supplement: Supplement 2. — Data Sharing Statement [file jamanetwopen-e2444593-s002.pdf]

Data Sharing Statement

Jiao. Fine Particulate Matter, Its Constituents, and Spontaneous Preterm Birth. *JAMA Netw Open*. Published online November 13, 2024. doi:10.1001/jamanetworkopen.2024.44593

Data
Data available: No

Additional Information
Explanation for why data not available: We use individual-level electronic health record data from a healthcare system; these data are confidential and protected by specific Institutional Review Board protocols.
